# Maternal emulsifier consumption programs offspring metabolic and neuropsychological health in mice

**DOI:** 10.1371/journal.pbio.3002171

**Published:** 2023-08-24

**Authors:** Maria Milà-Guasch, Sara Ramírez, Sergio R. Llana, Júlia Fos-Domènech, Lea Maria Dropmann, Macarena Pozo, Elena Eyre, Alicia G. Gómez-Valadés, Arnaud Obri, Roberta Haddad-Tóvolli, Marc Claret

**Affiliations:** 1 Neuronal Control of Metabolism (NeuCoMe) Laboratory, Institut d’Investigacions Biomèdiques August Pi i Sunyer (IDIBAPS), Barcelona, Spain; 2 CIBER de Diabetes y Enfermedades Metabólicas Asociadas (CIBERDEM), Barcelona, Spain; 3 School of Medicine, Universitat de Barcelona, Barcelona, Spain; INSERM, FRANCE

## Abstract

Modern lifestyle is associated with a major consumption of ultra–processed foods (UPF) due to their practicality and palatability. The ingestion of emulsifiers, a main additive in UPFs, has been related to gut inflammation, microbiota dysbiosis, adiposity, and obesity. Maternal unbalanced nutritional habits during embryonic and perinatal stages perturb offspring’s long–term metabolic health, thus increasing obesity and associated comorbidity risk. However, whether maternal emulsifier consumption influences developmental programming in the offspring remains unknown. Here, we show that, in mice, maternal consumption of dietary emulsifiers (1% carboxymethyl cellulose (CMC) and 1% P80 in drinking water), during gestation and lactation, perturbs the development of hypothalamic energy balance regulation centers of the progeny, leads to metabolic impairments, cognition deficits, and induces anxiety–like traits in a sex–specific manner. Our findings support the notion that maternal consumption of emulsifiers, common additives of UPFs, causes mild metabolic and neuropsychological malprogramming in the progeny. Our data call for nutritional advice during gestation.

## Abstract 2

Maternal consumption of emulsifiers present in ultra-processed food items has been linked to altered gut function and metabolism in their progeny. This study reveals that maternal emulsifier consumption during gestation and lactation lead to metabolic, cognitive and psychological malprogramming in the offspring.

## Introduction

Modern lifestyle promotes the disproportionate consumption of sugar and saturated fats together with a sedentary life, leading to the development of obesity (and its associated comorbidities), which has reached pandemic proportions [1]. In recent years, the so-called ultra-processed foods (UPFs) have become remarkably popular in the market due to their convenience and palatability. As defined by the NOVA classification, UPFs are industrial formulations with little or no whole food, poor nutritional quality, high glycemic load, low dietary fibers, and substantial amounts of additives (colorants, flavorings, sweeteners, thickeners, emulsifiers, etc.) [2]. Importantly, scientific evidence has associated UPF consumption with the development of obesity, type 2 diabetes (T2D), cardiovascular disease, cancer, depression, and gastrointestinal disorders [3–6].

Emulsifiers, one of the most common UPF additives, are used as stabilizers to form or maintain a homogenous mixture of 2 or more immiscible phases. They can be found in numerous UPF items, including margarines, mayonnaise, salad dressings, bread, ice creams, cake mixes, fruit juices, snacks, instant soups, and noodles among many others. The Food and Agriculture Organization/World Health Organization (FAO/WHO) allows the addition of emulsifiers up to 1%. Among the most used emulsifiers, sodium carboxymethyl cellulose (CMC), and polysorbate 80 (P80) have been extensively added to UPFs for over 30 years. Alarmingly, recent studies indicated that emulsifier consumption causes gut microbiota dysbiosis, intestinal inflammation and cancer, metabolic syndrome, and obesity [7–12].

Epidemiological and experimental evidence show that a perturbed environment during early life results in developmental adaptations that predispose the offspring to health disturbances in adulthood in both humans and rodents (“The Developmental Origins of Health and Disease” (DOHaD)) [13–16]. In this context, maternal dietary insults during gestation and lactation interfere with the programming of multiple neurocircuits [17–22], thus contributing to the development of diverse metabolic and neuropsychological disorders [23–25]. Indeed, it is worth noting that such pre- and perinatal nutritional challenges compromise the adequate development of hypothalamic feeding systems, including pro-opiomelanocortin (POMC) and agouti-related peptide (AgRP) neurons [19,20,26,27], which are crucial for systemic energy and metabolic homeostasis [28].

Within the context outlined above, the current study aimed to investigate the impact of emulsifier consumption during pregnancy and lactation on offspring’s long-term health using the mouse as an experimental model. Our data showed that maternal intake of emulsifiers induced mild metabolic and neuropsychological alterations in the progeny, thus calling for nutritional advice towards UPF consumption during gestation.

## Results

### Emulsifier consumption induces maternal glucose homeostasis disarrangements

**Fig 1:**
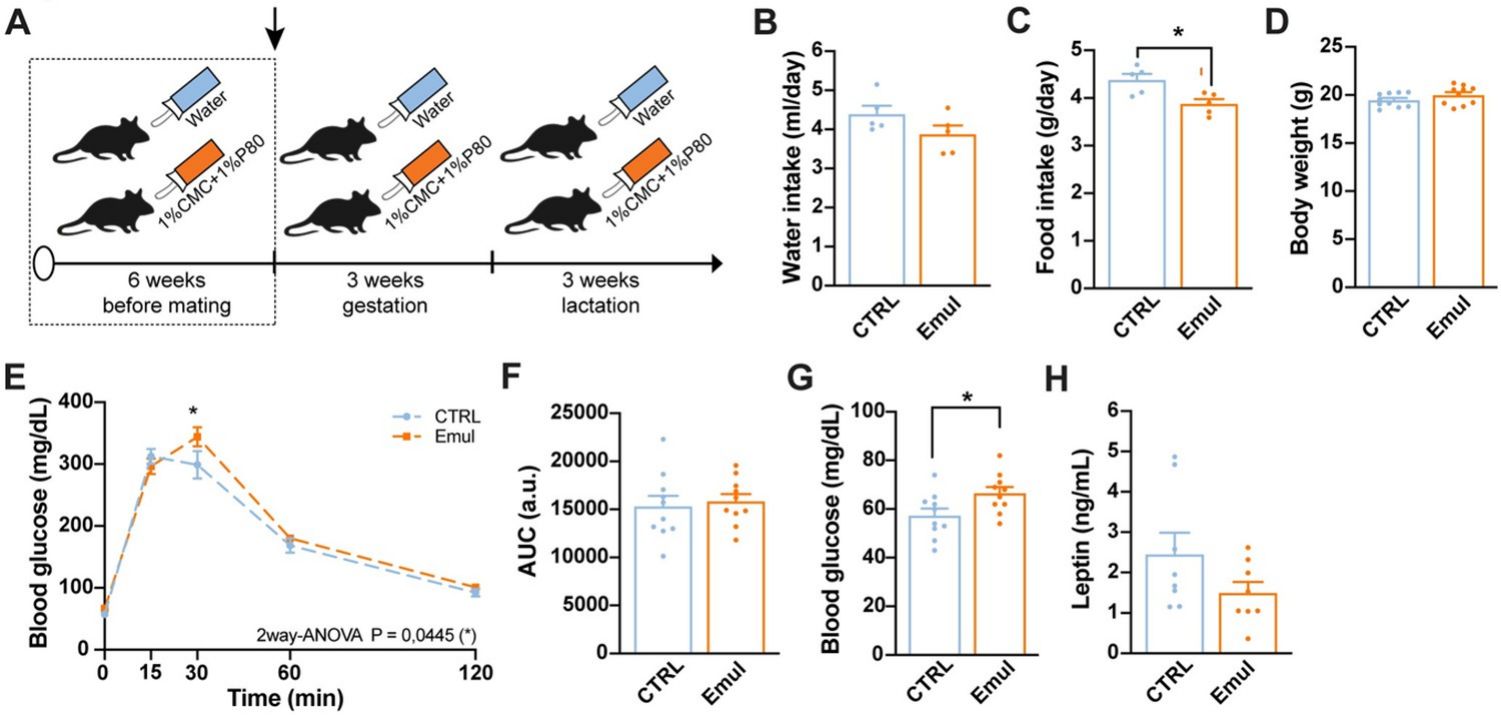
Emulsifiers induce mild glucose intolerance in female mice before the onset of pregnancy. (A) Experimental design of maternal emulsifier consumption highlighting the period of maternal characterization. (B) Daily water consumption of control and emulsifier–treated females before mating (*n* = 5/group). (C) Daily food intake of control and emulsifier–treated females before mating (*n* = 5/group). (D) Body weight of control and emulsifier–treated females before mating (*n* = 10 CTRL and *n* = 10 Emul). (E) GTT and (F) AUC of control and emulsifier females before mating (*n* = 10 CTRL and *n* = 10 Emul). (G) Fasting blood glucose levels of control and emulsifier–supplemented females before mating (*n* = 10 CTRL and *n* = 10 Emul). (H) Plasma leptin levels after 6 h in fasting of control and emulsifier–treated females before mating (*n* = 8 CTRL and *n* = 8 Emul). Data are derived from 1 single experiment. Data are expressed as mean ± SEM. Statistical analysis was performed with an unpaired *t* test in B, C, D, F, G, H, and by two–way ANOVA followed by Sidak’s post hoc analysis in E. *p < 0.05. The data underlying this figure can be found at DOI:10.6084/m9.figshare.22742759. AUC, area under the curve; GTT, glucose tolerance test.

To investigate the effects of maternal consumption of emulsifiers on offspring health, water (CTRL) or a mixture of 1% CMC and 1% P80 in drinking water (Emul) was provided to C57Bl/6 female mice for 6 weeks before pregnancy and throughout gestation and lactation (Fig 1A). Before the onset of pregnancy (after 6 weeks of emulsifier supplementation), CMC+P80 consumption did not affect the dams’ liquid intake (water or water supplemented with emulsifiers) (Fig 1B), but induced a slight decrease in daily food intake (Fig 1C) that did not reflect in changes in body weight (Fig 1D). However, emulsifier-treated females exhibited fasting hyperglycemia and presented mild glucose intolerance (Fig 1E–1G) with no changes in plasma leptin levels (Fig 1H). Continuous CMC+P80 exposure throughout pregnancy and lactation (S1A Fig) restored food intake (S1B Fig). Body weight (S1C Fig) and adiposity (S1D Fig) were unaltered. Glucose intolerance was intensified by the end of the treatment (S1E and S1F Fig), with no changes in basal glycemia (S1G Fig) and plasma leptin and insulin levels (S1H and S1I Fig). These results indicate that emulsifier consumption causes mild glucose homeostasis impairments in dams, thus altering the maternal environment during gestation and lactation.

### Maternal consumption of emulsifiers leads to mild metabolic impairments in the offspring at weaning in a sex-specific manner

**Fig 2:**
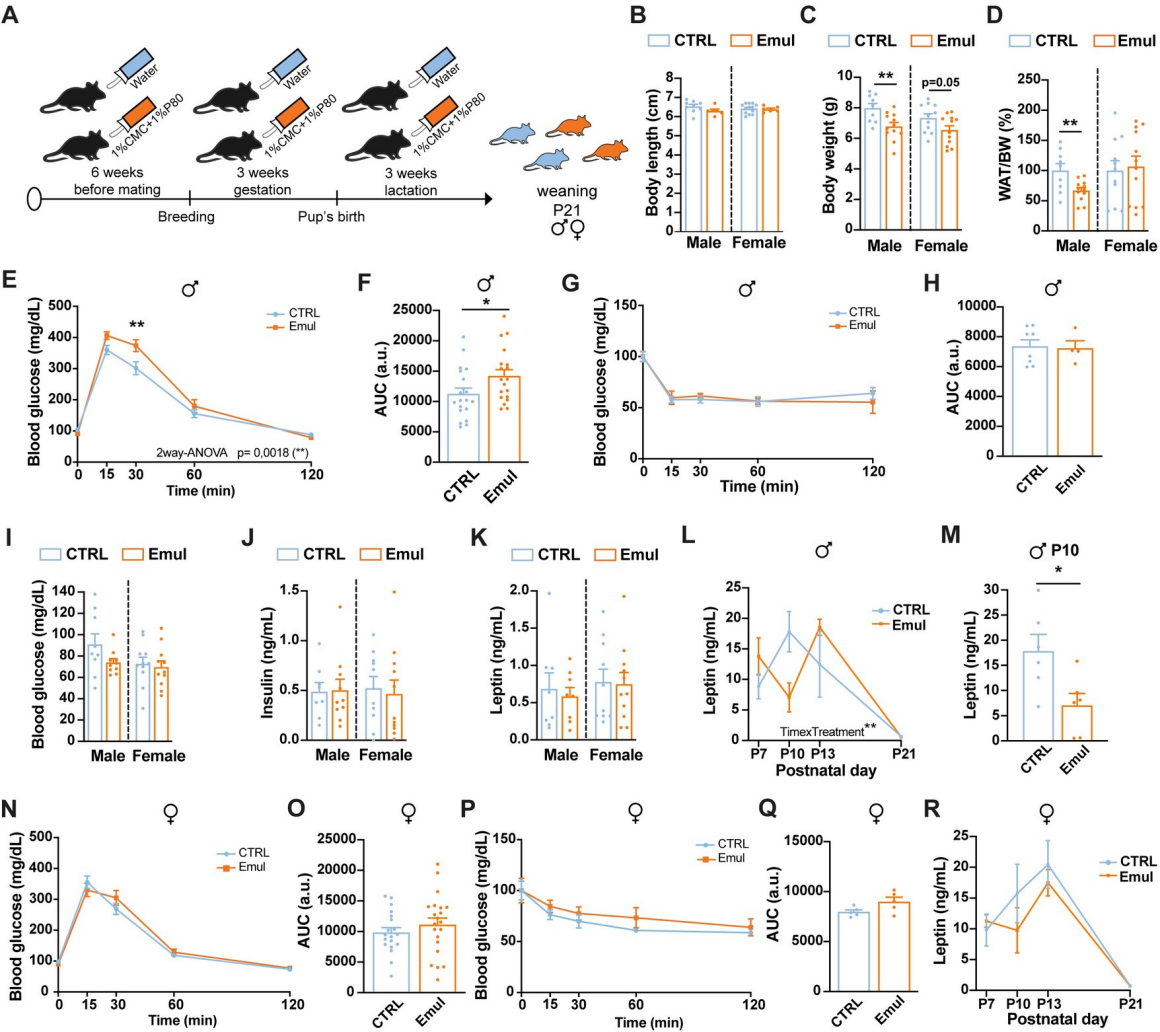
Maternal emulsifier consumption leads to mild metabolic impairments at weaning. (A) Experimental design of maternal emulsifier consumption and offspring collection at weaning. (B) Body length at weaning of male (*n* = 9 CTRL and *n* = 6 Emul) and female (*n* = 13 CTRL and *n* = 6 Emul) offspring from control and emulsifier–exposed dams. (C) Body weight at weaning of male (*n* = 9 CTRL and *n* = 12 Emul) and female (*n* = 11 CTRL and *n* = 12 Emul) offspring from control and emulsifier–exposed dams. (D) Epididymal and gWAT weight normalized by total body weight and represented as % of control animals in male (*n* = 9 CTRL and *n* = 12 Emul) and female (*n* = 11 CTRL and *n* = 12 Emul) offspring from control and emulsifier–exposed dams at weaning. (E) GTT and (F) AUC in male (*n* = 20 CTRL and *n* = 19 Emul) offspring from control and emulsifier–exposed dams at weaning. (G) ITT and (H) AUC in male (*n* = 8 CTRL and *n* = 4 Emul) offspring from control and emulsifier–exposed dams at weaning. (I) Six–hour fasting blood glucose levels in male (*n* = 9 CTRL and *n* = 10 Emul) and female (*n* = 11 CTRL and *n* = 11 Emul) offspring from control and emulsifier–exposed dams at weaning. (J) Plasma insulin levels in male (*n* = 8 CTRL and *n* = 10 Emul) and female (*n* = 10 CTRL and *n* = 11 Emul) offspring from control and emulsifier–exposed dams at weaning after 6 h of fasting. (K) Plasma leptin levels in male (*n* = 8 CTRL and *n* = 8 Emul) and female (*n* = 10 CTRL and *n* = 11 Emul) offspring from control and emulsifier–exposed dams at weaning after 6 h of fasting. (L) Plasma leptin levels across postnatal development (P7–P10–P13–P21) (P7 *n* = 6 CTRL and *n* = 5 Emul; P10 *n* = 6 CTRL and *n* = 6 Emul; P13 *n* = 6 CTRL and *n* = 6 Emul; P21 *n* = 8 CTRL and *n* = 8 Emul) in male offspring from control and emulsifier–exposed dams. (M) Peak plasma leptin levels at P10 (*n* = 6 CTRL and *n* = 6 Emul) in male offspring from control and emulsifier–exposed dams. (N) GTT and (O) AUC in female (*n* = 19 CTRL and *n* = 21 Emul) offspring from control and emulsifier–exposed dams at weaning. (P) ITT and (Q) AUC in female (*n* = 5 CTRL and *n* = 5 Emul) offspring from control and emulsifier–exposed dams at weaning. (R) Plasma leptin levels across postnatal development (P7–P10–P13–P21) (P7 *n* = 6 CTRL and *n* = 4 Emul; P10 *n* = 6 CTRL and *n* = 4 Emul; P13 *n* = 6 CTRL and *n* = 4 Emul; P21 *n* = 10 CTRL and *n* = 11 Emul) in female offspring from control and emulsifier–exposed dams. Data in B, G, H, L, M, P, Q, and R are derived from 1 single experiment. Data in C, D, E, F, I, J, K, N, and O are pools from 2 different experiments. Data are expressed as mean ± SEM. Statistical analysis was performed by unpaired *t* test in B, C, D, F, H, I, J, K, M, O, and Q and two–way ANOVA followed by Sidak’s post hoc analysis in E, G, N, and P. Panels L and R were analyzed using a two–way ANOVA mixed effects. *p < 0.05; **p < 0.01. The data underlying this figure can be found at DOI:10.6084/m9.figshare.22742759. AUC, area under the curve; GTT, glucose tolerance test; gWAT, gonadal white adipose tissue; ITT, insulin tolerance test.

We next assessed whether the consumption of emulsifiers during pregnancy and lactation impacted the metabolic health of the progeny (Fig 2A). Litter size was not altered upon maternal emulsifier consumption (CTRL: 7.4 ± 1.1 pups/litter; Emul: 8.8 ± 1.4 pups/litter, *t* test, *p* = 0.34). At weaning, male and female offspring from emulsifier-treated dams exhibited equivalent body lengths (Fig 2B) albeit were lighter (Fig 2C), and male mice showed decreased adiposity (Fig 2D). Despite this reduction in body weight and adiposity, male offspring from emulsifier-treated dams were mild glucose intolerant (Fig 2E and 2F) with no changes in insulin sensitivity (Fig 2G and 2H), glycemia or circulating insulin and leptin levels at weaning (Fig 2I–2K). Regardless comparable plasma leptin levels at weaning, male offspring from emulsifier-treated dams presented a delayed postnatal leptin surge that occurred at P13 instead of P10 (Fig 2L and 2M) [29]. No changes in overall metabolic parameters were observed in female offspring from emulsifier-treated dams (Fig 2I–2K and 2N–2R).

### Hypothalamic feeding-related determinants are altered upon maternal emulsifier consumption

**Fig 3:**
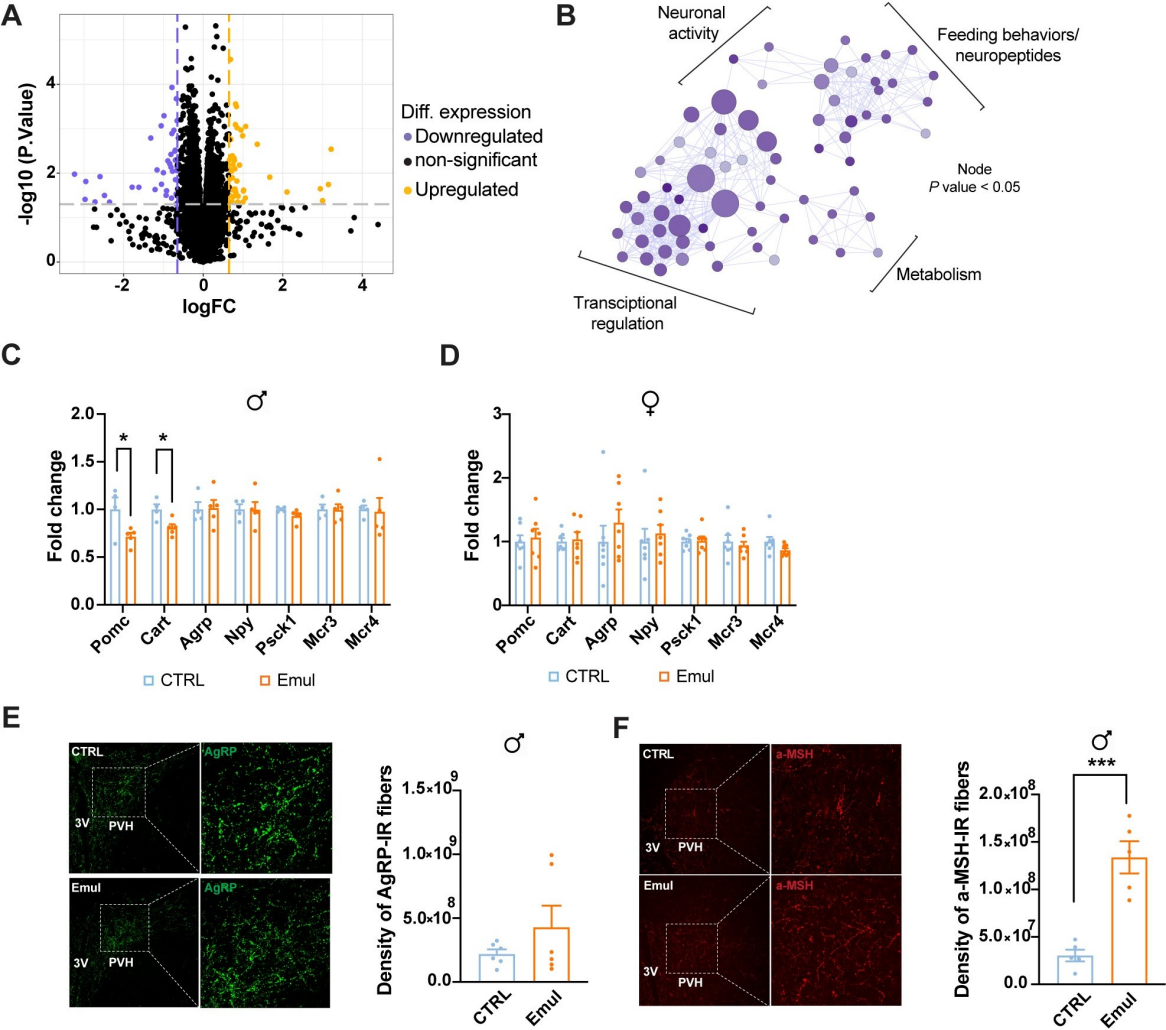
Hypothalamic feeding–related neuropeptides are altered upon maternal emulsifier consumption. (A) Volcano plot of transcript expression in the MBH between control and emulsifier offspring at weaning. Threshold for FC (±1.5) and FDR (*p* < 0.05) was considered. DEGs upon maternal emulsifier consumption are depicted in blue (down–regulated) and orange (up–regulated). Unchanged genes are represented in black (*n* = 4 CTRL and *n* = 5 Emul). (B) Cytoscape plot of the down–regulated enriched pathways (*p* < 0.05) in the offspring of emulsifier–exposed dams. (C) Transcript expression of orexigenic and anorexigenic peptides in the MBH in male offspring from control and emulsifier–exposed dams at weaning (*n* = 4 CTRL and *n* = 5 Emul). (D) Transcript expression of orexigenic and anorexigenic peptides in the MBH in female offspring from control and emulsifier–exposed dams at weaning (*n* = 7 CTRL and *n* = 7 Emul). (E) Representative immunofluorescence images showing AgRP staining density in the PVH of control and emulsifier male offspring at weaning and integrated density quantification (*n* = 6 mice/group). (F) Representative immunofluorescence images showing α–MSH staining density in the PVH of control and emulsifier male offspring at weaning and integrated density quantification (*n* = 5 mice/group). Data in C and D are derived from 1 single experiment. Data in **E** and **F** are pools from 2 different experiments. Data are expressed as mean ± SEM. Statistical analysis was performed by unpaired *t* test in C, D, E, and F. *p < 0.05; ***p < 0.001. The data underlying this figure can be found at DOI:10.6084/m9.figshare.22742759. *Pomc*, pro–opiomelanocortin; *Cart*, cocaine–and amphetamine–regulated transcript; *Agrp*, agouti–related peptide; *Npy*, neuropeptide Y; *Pcsk1*, proprotein convertase 1; *Mcr3*, melanocortin 3 receptor; *Mcr4*, melanocortin 4 receptor; α–MSH, alpha–melanocyte–stimulating hormone; PVH, paraventricular hypothalamic nucleus; 3V, third ventricle; DEG, differentially expressed gene; FC, fold change; FDR, false discovery rate; MBH, mediobasal hypothalamus.

Emulsifier consumption has been associated with changes in feeding-related neuropeptides in adult mice [11]. To understand the impact of maternal emulsifier consumption on hypothalamic development, we performed RNA sequencing (RNAseq) in the mediobasal hypothalamus (MBH) of male offspring at weaning. Principal component analysis (PCA) of individual samples identified 2 distinct clusters (S2A Fig). RNAseq analysis uncovered 83 differentially expressed genes (DEGs) upon maternal emulsifier consumption (Fig 3A). Out of the total amount of DEGs, 54% (45) of the genes were up-regulated and 46% (38) were down-regulated (Fig 3A and S1 Table). Enrichment pathway analysis of the down-regulated DEGs revealed significant changes in 4 main pathways: feeding behavior/neuropeptides, neuronal activity, metabolism, and transcriptional regulation (Fig 3B and S1 Table). To confirm that maternal consumption of emulsifiers affects the development of neuronal circuits controlling feeding behaviors, we analyzed the expression of key genes related to energy balance and food intake control in the MBH of the progeny of female mice exposed to emulsifiers during gestation and lactation. This analysis included the assessment of pro-opiomelanocortin (*pomc*), cocaine- and amphetamine-regulated transcript (*cart*), agouti-related peptide (*agrp*), neuropeptide Y (*npy*), proprotein convertase 1 (*pcsk1*), melanocortin 3 receptor (*mc3r*), and melanocortin 4 receptor (*mc4r*). Gene expression analysis revealed that maternal consumption of emulsifiers reduced *pomc* and *cart* expression at weaning exclusively in male offspring (Fig 3C and 3D), with no changes in the overall number and size of POMC neurons (S2B–S2D Fig).

Maternal dietary insults affect the development of axonal projections to target areas [19,26,30]. The changes observed in the expression of anorexigenic genes (*pomc* and *cart*) in males (Fig 3C) prompted us to evaluate if maternal emulsifier consumption would influence melanocortin projections to the paraventricular nucleus of the hypothalamus (PVH). While PVH AgRP staining density showed a non-significant trend to increase (Fig 3E), there was a notable increase in the density of α-melanocyte stimulating hormone (α-MSH, a bioactive anorexigenic product of POMC processing) staining in male offspring from emulsifier-treated dams at weaning (Fig 3F). These data suggested that maternal emulsifier consumption per se, without other components usually present in UPFs, is sufficient to induce a rewiring of the melanocortin hypothalamic feeding circuit that might underlie the metabolic changes observed in male offspring.

### Maternal emulsifier consumption leads to mild long-term metabolic impairments in male offspring

**Fig 4:**
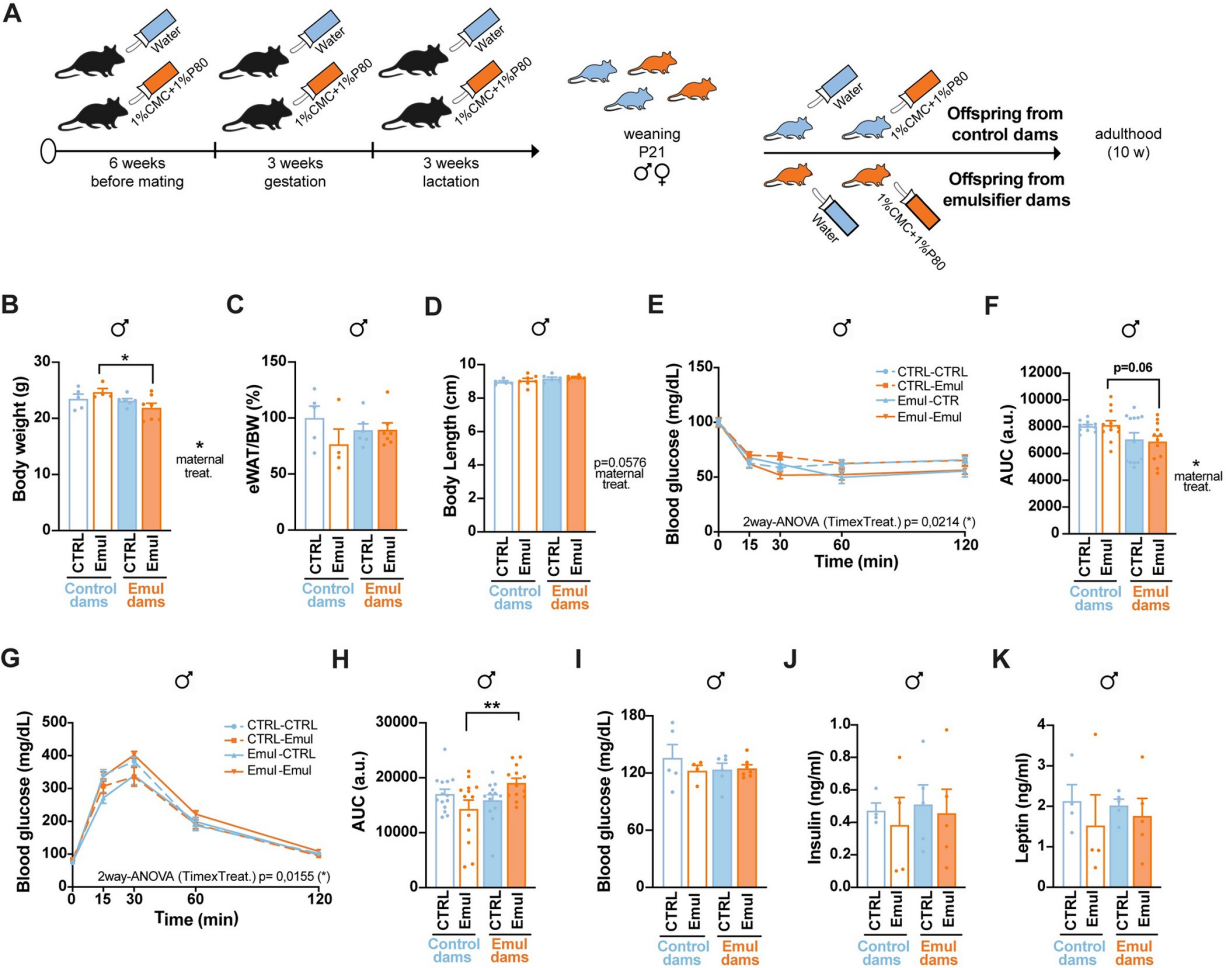
Maternal emulsifier consumption leads to mild long–term metabolic impairments in male offspring. (A) Schematic illustration of offspring treatment until adulthood (10 weeks of age). (B) Body weight at 10 weeks of age (*n* = 5 CTRL–CTRL; *n* = 4 CTRL–Emul; *n* = 6 Emul–CTRL; *n* = 7 Emul–Emul). (C) eWAT weight normalized by total body weight and represented as % of control animals at 10 weeks of age (*n* = 5 CTRL–CTRL; *n* = 4 CTRL–Emul; *n* = 6 Emul–CTRL; *n* = 7 Emul–Emul). (D) Body length at 10 weeks of age (*n* = 5 CTRL–CTRL; *n* = 7 CTRL–Emul; *n* = 6 Emul–CTRL; *n* = 5 Emul–Emul). (E) ITT and (F) AUC (*n* = 11 CTRL–CTRL; *n* = 12 CTRL–Emul; *n* = 13 Emul–CTRL; *n* = 12 Emul–Emul) at 10 weeks of age. (G) GTT and (H) AUC (*n* = 14 CTRL–CTRL; *n* = 13 CTRL–Emul; *n* = 13 Emul–CTRL; *n* = 13 Emul–Emul) at 10 weeks of age. (I) Six–hour fasting blood glucose levels (*n* = 5 CTRL–CTRL; *n* = 4 CTRL–Emul; *n* = 6 Emul–CTRL; *n* = 7 Emul–Emul) at 10 weeks of age. (J) Plasma insulin levels after 6 h of fasting at 10 weeks of age (*n* = 4 CTRL–CTRL; *n* = 4 CTRL–Emul; *n* = 5 Emul–CTRL; *n* = 5 Emul–Emul). (K) Plasma leptin levels after 6 h of fasting at 10 weeks of age (*n* = 4 CTRL–CTRL; *n* = 4 CTRL–Emul; *n* = 5 Emul–CTRL; *n* = 5 Emul–Emul). Data in B, C, D, I, J, and K are derived from 1 single experiment. Data in E, F, G, and H are pools from 2 different experiments. Data are expressed as mean ± SEM. Statistical analysis was performed by two–way ANOVA followed by Sidak’s post hoc analysis. *p < 0.05; **p < 0.01. The data underlying this figure can be found at DOI:10.6084/m9.figshare.22742759. AUC, area under the curve; eWAT, epididymal white adipose tissue; GTT, glucose tolerance test; ITT, insulin tolerance test.

Next, we interrogated whether early life exposure to CMC+P80 induces long-term metabolic health disruptions. To differentiate between maternal programming consequences and emulsifier consumption after birth, we divided control and emulsifier offspring into 4 groups at weaning: maternal control + water (CTRL—CTRL), maternal control + emulsifiers after weaning (CTRL—Emul), maternal emulsifier + water (Emul—CTRL), and maternal emulsifier + emulsifier (Emul—Emul) treatment after weaning until 10 weeks of age (Fig 4A). In males, maternal consumption of emulsifiers, independent of its exposure after weaning, led to body weight reduction (Fig 4B), without significant changes in adiposity (Fig 4C) or body length (Fig 4D). Male offspring born from dams treated with emulsifiers showed higher insulin sensitivity (Fig 4E and 4F), without changes in food intake (S4A Fig), glucose tolerance, blood glucose, insulin, and leptin levels (Fig 4G–4K). Of note, life-long exposure to emulsifiers (intrauterine and postnatal life) induced stronger glucose homeostasis impairments leading to glucose intolerance (Fig 4H) (maternal control + emulsifier: 14,326 ± 5,834 area under the curve (AUC) versus maternal emulsifier + emulsifier: 19,084 ± 3,039 AUC, *p* = 0.0099). These effects were sex-specific since female offspring from emulsifier-treated dams did not show alterations in any of the analyzed parameters (S3A–S3J and S4B Figs). The delayed leptin surge seen in male offspring from emulsifiers-treated dams (Fig 2L and 2M) prompted us to investigate their ability to respond to the anorexigenic effects of leptin. Maternal emulsifier consumption, or emulsifier consumption during life, did not perturb the weight- and food intake-reducing effects of exogenously administered leptin (S4C and S4D Fig). These results suggest that maternal consumption of emulsifiers affects the metabolic programming of male offspring, triggering mild alterations in glucose metabolism in adulthood.

### The combination of emulsifiers and western diet does not exacerbate the metabolic impairments derived from maternal programming

**Fig 5:**
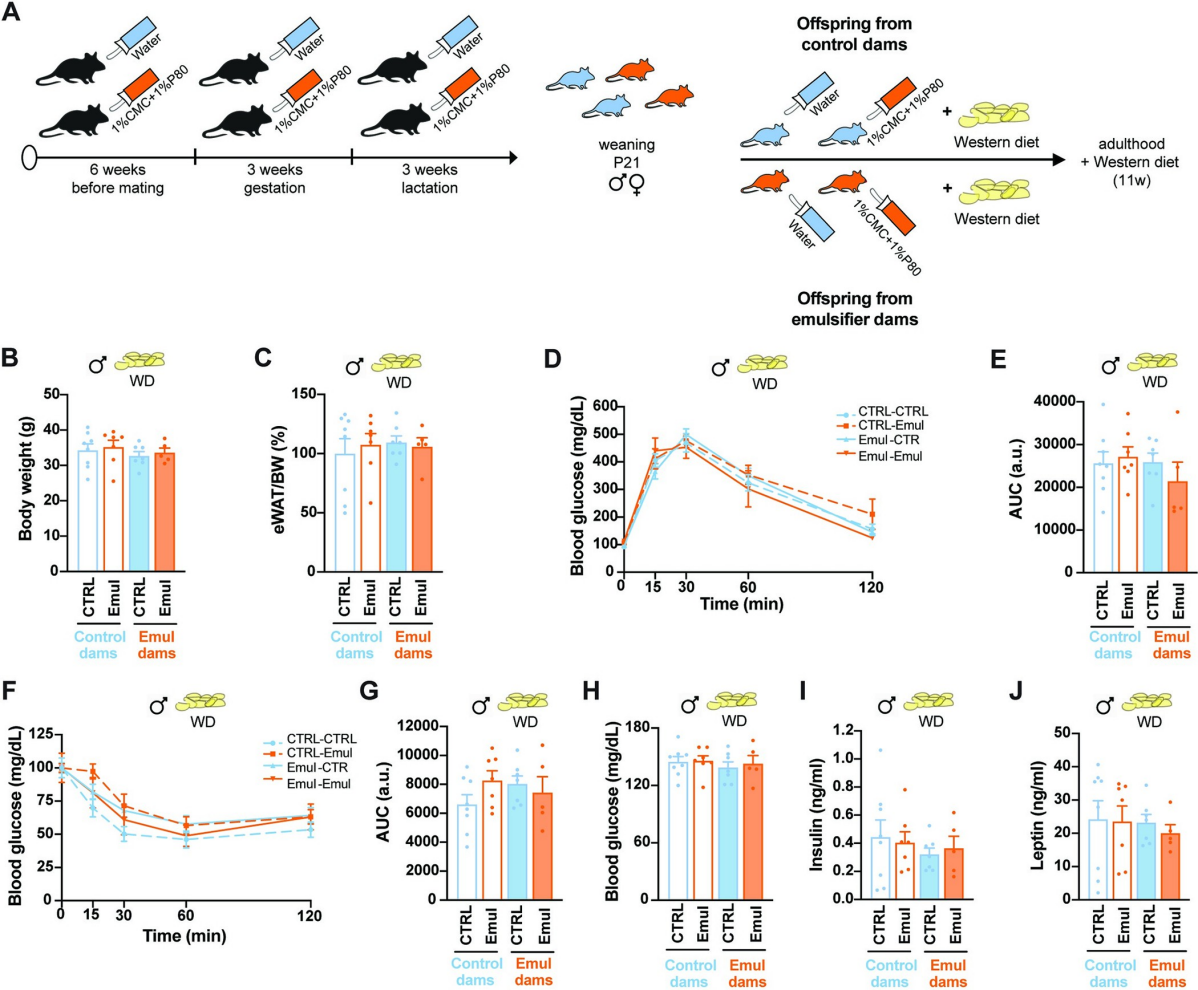
Metabolic impairments derived from western diet consumption are not exacerbated in male offspring from emulsifier–treated dams. (A) Schematic illustration of offspring treatment until adulthood. (B) Body weight at 22 weeks of age, after 11 weeks of WD exposure (*n* = 8 CTRL–CTRL; *n* = 7 CTRL–Emul; *n* = 7 Emul–CTRL; *n* = 5 Emul–Emul). (C) eWAT weight normalized by total body weight and represented as % of control animals at 22 weeks of age, after 11 weeks of WD exposure (*n* = 8 CTRL–CTRL; *n* = 7 CTRL–Emul; *n* = 7 Emul–CTRL; *n* = 5 Emul–Emul). (D) GTT and (E) AUC (*n* = 8 CTRL–CTRL; *n* = 7 CTRL–Emul; *n* = 7 Emul–CTRL; *n* = 5 Emul–Emul) at 19 weeks of age, after 8 weeks of WD exposure. (F) ITT and (G) AUC (*n* = 8 CTRL–CTRL; *n* = 7 CTRL–Emul; *n* = 7 Emul–CTRL; *n* = 5 Emul–Emul) at 19 weeks of age, after 8 weeks of WD exposure. (H) Six–hour fasting blood glucose levels at 22 weeks of age, after 11 weeks of WD exposure (*n* = 8 CTRL–CTRL; *n* = 7 CTRL–Emul; *n* = 7 Emul–CTRL; *n* = 5 Emul–Emul). (I) Plasma insulin levels after 6 h of fasting at 22 weeks of age, after 11 weeks of WD exposure (*n* = 8 CTRL–CTRL; *n* = 7 CTRL–Emul; *n* = 7 Emul–CTRL; *n* = 5 Emul–Emul). (J) Plasma leptin levels after 6 h of fasting at 22 weeks of age, after 11 weeks of WD exposure (*n* = 8 CTRL–CTRL; *n* = 7 CTRL–Emul; *n* = 7 Emul–CTRL; *n* = 5 Emul–Emul). Data are derived from 1 single experiment. Data are expressed as mean ± SEM. Statistical analysis was performed by two–way ANOVA followed by Sidak’s post hoc analysis. The data underlying this figure can be found at DOI:10.6084/m9.figshare.22742759. AUC, area under the curve; eWAT, epididymal white adipose tissue; GTT, glucose tolerance test; ITT, insulin tolerance test; WD, western–style diet.

**Fig 6:**
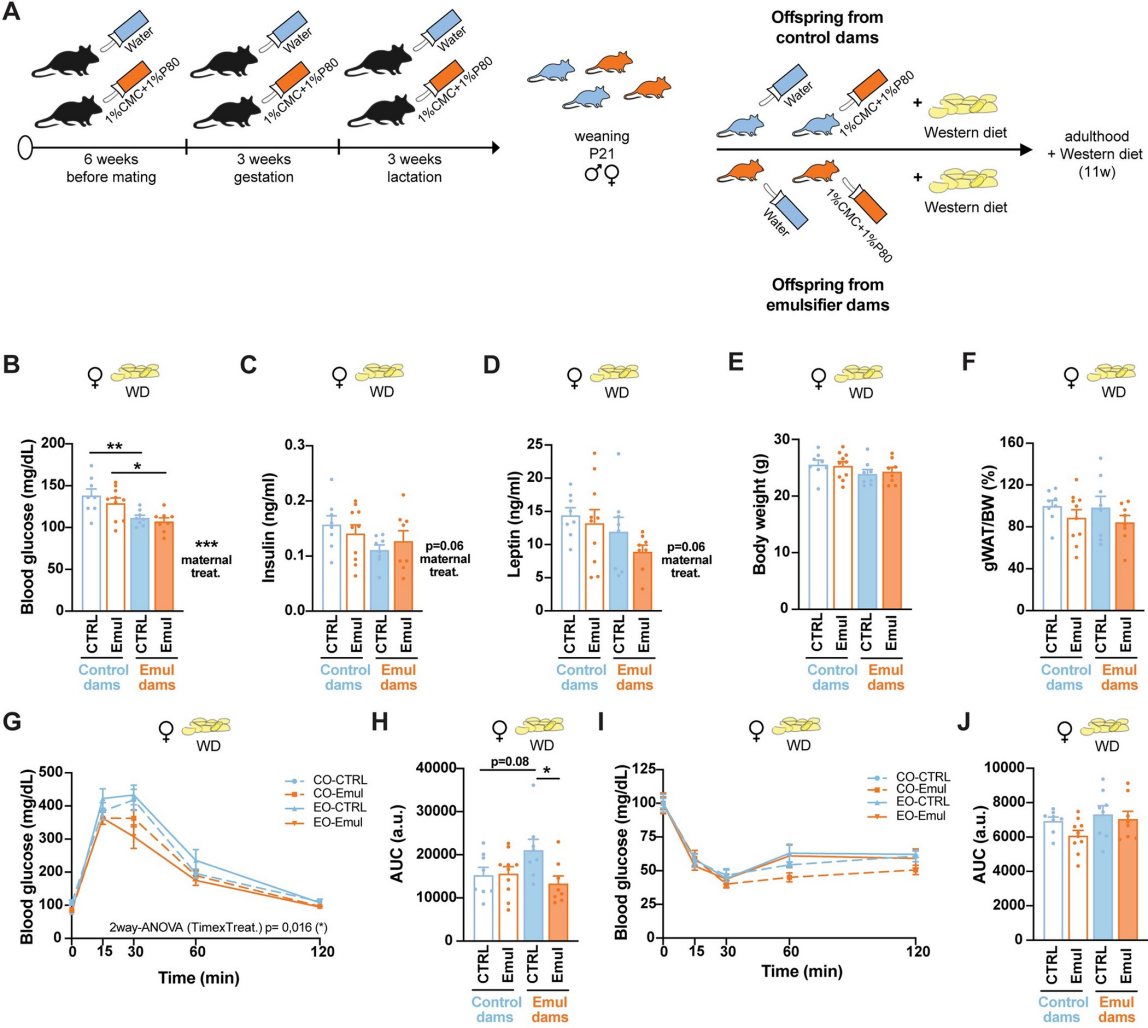
Metabolic outcomes derived from western diet consumption on female offspring from emulsifier–treated dams. (A) Schematic illustration of offspring treatment until adulthood. (B) Six–hour fasting blood glucose levels at 22 weeks of age, after 11 weeks of WD exposure (*n* = 8 CTRL–CTRL; *n* = 10 CTRL–Emul; *n* = 8 Emul–CTRL; *n* = 8 Emul–Emul). (C) Plasma insulin levels after 6 h of fasting at 22 weeks of age, after 11 weeks of WD exposure (*n* = 8 CTRL–CTRL; *n* = 10 CTRL–Emul; *n* = 8 Emul–CTRL; *n* = 8 Emul–Emul). (D) Plasma leptin levels after 6 h of fasting at 22 weeks of age, after 11 weeks of WD exposure (*n* = 8 CTRL–CTRL; *n* = 10 CTRL–Emul; *n* = 8 Emul–CTRL; *n* = 8 Emul–Emul). (E) Body weight at 22 weeks of age, after 11 weeks of WD exposure (*n* = 8 CTRL–CTRL; *n* = 10 CTRL–Emul; *n* = 8 Emul–CTRL; *n* = 8 Emul–Emul). (F) gWAT weight normalized by total body weight and represented as % of control animals at 22 weeks of age, after 11 weeks of WD exposure (*n* = 8 CTRL–CTRL; *n* = 10 CTRL–Emul; *n* = 8 Emul–CTRL; *n* = 8 Emul–Emul). (G) GTT and (H) AUC (*n* = 8 CTRL–CTRL; *n* = 10 CTRL–Emul; *n* = 8 Emul–CTRL; *n* = 8 Emul–Emul) at 19 weeks of age, after 8 weeks of WD exposure. (I) ITT and (J) AUC (*n* = 8 CTRL–CTRL; *n* = 10 CTRL–Emul; *n* = 8 Emul–CTRL; *n* = 8 Emul–Emul) at 19 weeks of age, after 8 weeks of WD exposure. Data are derived from 1 single experiment. Data are expressed as mean ± SEM. Statistical analysis was performed by two–way ANOVA followed by Sidak’s post hoc analysis. *p < 0.05; **p < 0.01; ***p < 0.001. The data underlying this figure can be found at DOI:10.6084/m9.figshare.22742759. AUC, area under the curve; GTT, glucose tolerance test; gWAT, gonadal white adipose tissue; ITT, insulin tolerance test; WD, western–style diet.

Emulsifiers are mostly present in UPFs, which are highly rich in carbohydrates and fats. We then wondered if the metabolic programming effects due to maternal consumption of emulsifiers would be accentuated once challenged with a high-fat high-sucrose western-style diet (WD). To this end, offspring from control and emulsifier-treated dams were exposed to WD for 11 weeks (Fig 5A). Surprisingly, the combination of emulsifiers and WD did not exacerbate any of the metabolic changes observed in male offspring (Fig 5B–5J). In females, concomitant exposure to maternal emulsifiers and WD access during adulthood (Fig 6A) lowered fasting glucose levels (Fig 6B) and decreased diet-induced hyperinsulinemia and hyperleptinemia (Fig 6C and 6D), while showing similar body weight (Fig 6E) and adiposity (Fig 6F) as control counterparts. Furthermore, WD-fed females born to emulsifier-treated dams with continuous exposure to emulsifier were less glucose intolerant (Fig 6G and 6H) (maternal emulsifier + water: 21,114 ± 7,020 AUC versus maternal emulsifier + emulsifier: 13,350 ± 4,885 AUC, *p* = 0.0178) with no changes in insulin sensitivity (Fig 6I and 6J).

### Maternal emulsifier consumption disrupts offspring neuropsychological health

**Fig 7:**
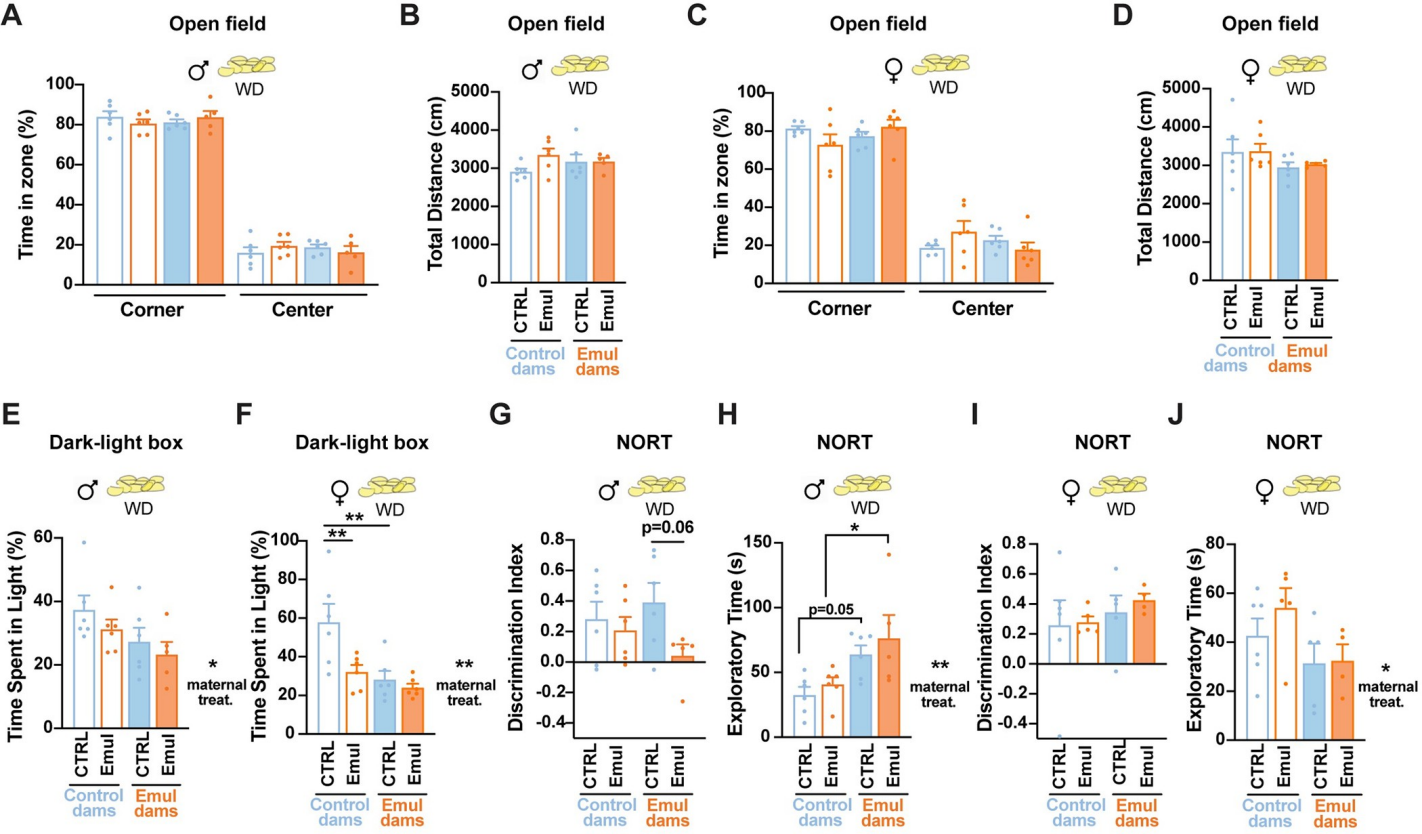
Maternal emulsifier consumption disrupts offspring neuropsychological health. (A–D) Open field performance in 23–week–old male (A and B) (*n* = 6 CTRL–CTRL; *n* = 6 CTRL–Emul; *n* = 6 Emul–CTRL; *n* = 5 Emul–Emul) and female (C and D) (*n* = 6 mice/group) offspring born of control and emulsifier–exposed mothers, including time spent per zone (A and C) and total distance traveled (B and D) after 12 weeks of WD exposure. (E, F) Time spent in the light compartment during the dark–light box test in 23–week–old male (E) (*n* = 6 CTRL–CTRL; *n* = 6 CTRL–Emul; *n* = 6 Emul–CTRL; *n* = 5 Emul–Emul) and female (F) (*n* = 6 mice/group) offspring born of control and emulsifier–exposed mothers after 12 weeks of WD exposure. (G–J) Short–term memory parameters in 24–week–old male (G and H) (*n* = 6 CTRL–CTRL; *n* = 6 CTRL–Emul; *n* = 6 Emul–CTRL; *n* = 5 Emul–Emul) and female (I and J) (*n* = 6 CTRL–CTRL; *n* = 5 CTRL–Emul; *n* = 5 Emul–CTRL; *n* = 4 Emul–Emul) offspring born of control and emulsifier–exposed mothers, after 13 weeks of WD exposure, including discrimination index (G and I) and exploratory time (H and J). Data are derived from 1 single experiment. Data are expressed as mean ± SEM. Statistical analysis was performed by two–way ANOVA followed by Sidak’s post hoc analysis. *p < 0.05; **p < 0.01. The data underlying this figure can be found at DOI:10.6084/m9.figshare.22742759. WD, western–style diet.

CMC or P80 consumption have been shown to cause anxiety-like behaviors [11]. We next asked if maternal consumption of emulsifiers was sufficient to induce neuropsychological deficits in the offspring. To test this, we conducted a behavioral screening of anxiety-like phenotypes by exposing the offspring to the open field and dark-light box paradigms. Offspring of emulsifier-treated dams presented no changes in locomotor activity in an open field test (S5A–S5D Fig) but male offspring exhibited a significant decrease in the time spent in the light compartment of a dark-light box paradigm (S5E and S5F Fig). Anxiety-like behaviors were intensified upon WD challenge during adulthood, particularly in females (Fig 7A–7F). These results suggested increased anxiety-like states in the offspring of dams exposed to CMC+P80 during gestation and lactation. In addition, dietary insults during pregnancy have been linked to cognitive dysfunction in the offspring [25,31]. While cognition was not impaired in the offspring born to emulsifier-treated dams fed with normal chow diet (S5G–S5J Fig), life-long exposure to emulsifiers in combination with WD led to cognitive impairments in a novel object recognition test (NORT) in males (Fig 7G and 7H). Female offspring did not present memory recognition deficits (Figs 7I and 7J and S5I and S5J). Together, these results demonstrate that emulsifier consumption during pregnancy could induce life-long consequences in offspring neuropsychological and metabolic health.

## Discussion

The consumption of UPFs has increased sharply over the last 2 decades. In fact, recent surveys have shown that UPF consumption contributes to 25% to 50% of the total daily caloric intake in adults [32] and more than 60% among school-age children in the United Kingdom and the United States [33,34]. UPF intake during pregnancy and its potential adverse effects on maternal–child health have started to be investigated in humans, identifying a positive association between UPF consumption and gestational weight gain, neonatal adiposity, and the development of attention deficit hyperactivity disorder [35–38]. The combination of distinct additives (emulsifiers, sweeteners, colorants, flavorings, etc.) concur with saturated fats and sugars in UPFs and, therefore, the precise contribution of each of these additives to maternal health and offspring developmental programming needs to be carefully assessed. In the present study, we investigated the transgenerational health impact of emulsifiers in mice. In our experimental model, dams and/or offspring mice were exposed to prolonged and continuous amount of CMC+P80, 2 extensively used emulsifiers in the UPF industry. This pattern of administration echoes to some extent the human environment, where exposure to emulsifiers is high and usually combined with other additives, although its consumption occurs in an intermittent manner. Given that access to UPF occurs throughout life (and not only during pregnancy and breastfeeding), we opted for an extended experimental design by providing CMC+P80 several weeks before fecundation. This approach considered the potential long-term effects of emulsifiers on the female intrauterine environment. We found that the maternal consumption of these emulsifiers was sufficient to induce mild metabolic, cognitive, and psychological impairments in male offspring (and to a lesser extent in females).

As regards metabolism, the mild decrease in food intake observed in the female mice before the onset of pregnancy (after 6 weeks of CMC+P80 treatment) disappeared upon prolonged exposure to emulsifiers. This could account to an adaptative response after short-term supplementation with emulsifiers. Our results also indicated that maternal ingestion of emulsifiers resulted in glucose intolerance, even in the absence of body weight gain. In addition, maternal emulsifier consumption delayed the postnatal leptin surge in male offspring. Alterations in leptin levels and glycemic fluctuations during pregnancy can disrupt neuronal specification, proliferation and wiring of hypothalamic circuits in the offspring. Indeed, leptin levels directly influence axonal outgrowth of POMC and AgRP neurons during lactation [23,39]. Therefore, the delayed leptin surge in male offspring of dams exposed to CMC+P80 could underlie the alterations in α-MSH staining innervating the PVH at weaning. Nevertheless, the leptin system seems to function correctly as plasma leptin levels and leptin response were not compromised in adult offspring.

Moreover, our gene expression and α-MSH staining density analysis suggested that anorexigenic brain circuits were more sensitive to the detrimental effects of emulsifier consumption during pregnancy. In addition, the reduction in *pomc* and *cart* expression in male offspring could reflect a compensatory mechanism for the higher α-MSH staining density reaching the PVH. This could underlie the decrease in body weight and adiposity observed in these animals at weaning and during adulthood. Together, our results suggest that emulsifiers can influence the development of hypothalamic neurocircuits during early life in a similar fashion as variations in nutrients do, including glucose, lipids, and food additives [40–42].

Emerging evidence suggests, in both mice and humans, that low concentrations of dietary emulsifiers are sufficient to impact intestinal barrier function and to induce gut inflammation, thus increasing the incidence of intestinal diseases and metabolic syndrome [8,43–46]. The fact that emulsifiers can modulate the gut microbiome [8,47], perturbing its function and generating inflammation, could also contribute to the metabolic impairments and cognition deficits observed in male offspring. In this context, maternal consumption of P80 during gestation and lactation leads to gut dysbiosis and predisposes to colitis in the offspring [48]. Moreover, dysbiosis of the maternal gut microbiome during pregnancy has been shown to modulate fetal thalamocortical axonogenesis [49], disrupt brain function and behavior in the offspring [50–52]. Alterations in gut microbiome could also play a part in the anxiety-like traits we observed in both sexes since emulsifier consumption has been linked to anxiety-like behavior in mice [11]. These observations suggest that emulsifiers may also interfere with the development of other brain regions related with diverse behavioral processes.

Our results also indicated that maternal emulsifier consumption had a sex-specific effect in the offspring. Indeed, male offspring were more susceptible to metabolic and neuropsychological disruptions caused by maternal emulsifier consumption, providing additional evidence that males and females respond differently to a suboptimal maternal environment [21,53]. In addition, at a first glance, the combined exposure of emulsifiers and western diet during adulthood seems to alleviate female mice from the effects of energy-dense diets on glucose metabolism. This alteration, however, could be the consequence of impaired glucose absorption by the gut, increased glycosuria or defective nutrient transport throughout the intestinal cavity. The sex-related developmental mechanisms underlying these metabolic and cognitive sexual dimorphisms require further investigation.

The effects of emulsifier ingestion on maternal programming were stronger than those from exposure after weaning, agreeing with the idea that intrauterine and early postnatal life are critical developmental periods that, if disrupted, can have profound metabolic consequences in adulthood. Some of these effects (e.g., glucose tolerance, cognition) seemed to be slightly worsen upon prolonged exposure to emulsifiers (maternal plus after weaning treatments). Whether the alterations in offspring metabolic and neuropsychological health arise from disturbances induced by emulsifiers in the mothers before pregnancy onset or directly derive from effects during gestation and lactation could not be addressed in the present study. Follow-up studies analyzing the consequences of emulsifiers at each stage separately would be necessary. In addition, how long-term emulsifiers (mimicking persistent exposure to UPFs throughout life) affect female reproductive status, embryonic survival as well as maternal and offspring health outcomes demand further evaluation.

Previous studies have showed that consumption of CMC or P80 individually during adulthood was sufficient to induce metabolic alterations and microbiota dysbiosis in mice [8,11]. However, mice from control dams exposed to CMC+P80 did not show metabolic alterations under our experimental settings. This discrepancy with previous studies may be due to differences in the experimental protocol implemented, as our approach differed in the onset and length of treatment as well as in the combination of compounds. We combined these 2 broadly used emulsifiers in order to maximize their effects and mimic, to some extent, the presence of diverse emulsifiers in most contemporary processed food items. It is plausible that the mixture of CMC+P80 induces a milder (rather than an additive or synergistic) effect, but this requires further investigation.

Food packaging labels provide null or scarce information regarding the actual content of additives in UPFs. This greatly limits consumer knowledge regarding the levels eaten and our ability to avoid the ingestion of a large and diverse array of food additives [32,54]. Even food items that are perceived as “healthy,” such as vegan/vegetarian products, contain large amounts of additives (including emulsifiers) that per se could induce long-term metabolic impairments. In this regard, it is important to bring about greater societal consciousness that some apparently “healthy” industrial formulations might induce metabolic alterations to a similar extent as products usually considered “unhealthy.”

Collectively, our study showed that maternal consumption of emulsifiers commonly present in UPF items induced mild metabolic, cognitive, and psychological programming effects in the offspring in a sex-dependent manner. Our findings emphasize the importance of a healthy developmental environment during gestation and endorse the idea that the amount of UPFs consumed during gestation should be taken into serious consideration. We call for awareness of UPF intake during pregnancy and lactation to avoid potential detrimental effects on the metabolic and neuropsychiatric health of the progeny, thus building adequate nutritional habits for mothers and infants.

## Materials and methods

### Ethics statement

All animal procedures were performed according to the Spanish Policy for Animal Protection RD53/2013, which complies with the European Union Directive 2010/63 on the protection of animals used for experimental and other scientific purposes. The protocol was approved by the Animal Experimentation Ethics Committee from the University of Barcelona (protocol number: 00346–22) and performed by accredited personnel.

### Animal care, mouse lines, and diets

In-house bred C57BL/6 mice were maintained on a temperature-controlled, 12-h light/dark cycle with free access to standard chow diet (Teklad maintenance diet 14% protein; Envigo). In specific studies, western diet (40% kcal from fat and 43% kcal from carbohydrates; Research Diets) was provided ad libitum to the offspring for 11 weeks (starting at 10 weeks of age). The age and number of mice analyzed per experiment are detailed in the figure legends. All animal studies were performed with the approval of the University of Barcelona Ethics Committee, complying with current Catalan, Spanish and European legislation.

### Mouse breeding, offspring, and emulsifier treatment

Wild-type C57Bl/6 female mice of 6 to 7 weeks of age fed with standard diet were exposed to either drinking water without any supplement (maternal control) or supplemented with emulsifiers (maternal emulsifiers): sodium carboxymethyl cellulose (CMC; 41931, Sigma) and polysorbate 80 (P80; W291706, Sigma) (1% each in drinking water). These solutions were changed weekly. After 6 weeks of treatment, female mice were crossed with chow-fed wild-type C57Bl/6 male mice. Weight gain was measured weekly to confirm pregnancies. Litter size was adjusted (between postnatal day (P)5-P7) to 6–8 pups to ensure adequate and standardized nutrition until weaning. Dams were kept with their offspring until weaning at P21.

After weaning, the offspring were subdivided into control (drinking water without supplementation) and emulsifier group (CMC+P80; 1% in drinking water), ending up with 4 different conditions: maternal control—offspring control (CTRL–CTRL), maternal control—offspring emulsifiers (CTRL–Emul), maternal emulsifiers—offspring control (Emul–CTRL), and maternal emulsifiers—offspring emulsifiers (Emul–Emul).

This experimental plan has the advantage of clearly differentiating the effects of emulsifiers derived from maternal programming from post-weaning stages. This way, we offer a wider view of the impact of these food additives during different life periods on diverse health aspects. A limitation of this design is that we cannot rule out direct exposure of emulsifiers during perinatal life (P0-P21).

### Food and water intake measurements

For food and water intake studies, mice were singly housed and acclimatized for 1 week. Daily food intake (in grams) was manually measured for 5 consecutive days using a precision scale. Water intake (water or water supplemented with emulsifiers) was assessed by the total daily liquid intake (in mL) for 5 consecutive days.

### Offspring body length

Body length (in cm) was measured with a ruler from the nose to the base of the tail at weaning (P21) and adulthood. A total number of 4 control litters and 5 emulsifier-treated litters were used. The same cohorts were followed from weaning until adulthood.

### Physiological measurements

Body weights and gonadal fat pads were monitored using a precision scale. For metabolic studies, the same cohorts were followed from weaning until adulthood. A total number of 11 control litters and 10 emulsifier-treated litters were used for metabolic studies. For the glucose tolerance test (GTT), mice were intraperitoneally (IP) injected with a single bolus of D-glucose (2 g/kg) after 6 h (P21 animals) or overnight (adult mice) fasting. For insulin sensitivity tests, mice were IP injected with a single bolus of insulin (0.3 mU/kg during weaning, 0.4 mU/kg for adults on chow diet, 0.5 mU/kg for adults on western diet) after 6 h fasting. Blood glucose levels were measured using a glucometer (Nova Pro Biomedical) 0, 15, 30, 60, and 120 min after glucose/insulin administration. Plasma insulin and leptin levels were measured after 6 h of fasting using commercially available enzyme-linked immunosorbent assay (ELISA) kits (Crystal Chem).

### Leptin surge assay

Male and female offspring from control (*n* = 6) or emulsifier-treated (*n* = 9) dams were culled on P7, P10, P13, and P21, and trunk blood was collected. Leptin levels in the plasma were assayed using commercially available ELISA kits (Crystal Chem).

### Leptin sensitivity test

Mice were singly housed and acclimatized by subjecting them to handling and sham injections for 1 week prior to study. Leptin tests were conducted in a crossover fashion. Twelve-week-old mice from each experimental group were IP injected with either 5 μg/g of mouse leptin (R&D Systems) or vehicle 1 h before lights out (7 PM). Food intake and body weights were recorded next morning (9 AM). A total number of 5 control litters and 4 emulsifier-treated litters were used.

### RNA preparation and sequencing

Total RNA samples from P21 male mice (4 control litters and 5 emulsifier-treated litters) were processed at the IDIBAPS genomics’ platform for Tapestation quality control and subsequent sequencing. All samples had an RNA integrity number (RIN) > 8. mRNA strand-specific RNA libraries were generated using 200 ng of total RNA using the Stranded mRNA Prep Ligation kit (Illumina) following the manufacturer’s instructions. Libraries were sequenced on an Illumina NextSeq2000 (Illumina) in paired-end mode with a read length of 2 × 50 bp. Around 40 million of paired-end reads were generated for each sample/condition.

### RNA sequencing analysis

FastQC analysis was used to assess the quality of sequence readings (https://www.bioinformatics.babraham.ac.uk/projects/fastqc/). Transcript quantification for each sample was accomplished using Kallisto [55] on the mouse reference genome mm10. Only genes with a cpm >1 in at least 4 samples were considered. Limma package was used to perform differential gene expression analysis [56]. Genes with a fold change higher/lower than 1.5 and a *p*-value less than 0.05 were considered significant. Pathway enrichment analysis on differentially expressed genes was performed on g:Profiler [57]. Cytoscape was used to create enrichment maps [58].

### Quantitative polymerase chain reaction (qPCR)

MBH samples derived from 14 different litters (*n* = 7 litters/maternal treatment) were harvested, immediately frozen in liquid nitrogen, and stored at −80°C. Tissues were homogenized and total mRNA was isolated using Trizol (Invitrogen). RNA products were reverse transcribed using reagents from Applied Biosystems. Quantitative PCR was conducted using Premix Ex Taq master mix (Takara) in an ABI Prism 7900 HT system (Applied Biosystems). Taqman Gene Expression assay FAM/TAMRA probes (Applied Biosystems) used for qPCR analysis were: *Pomc* (Mm00435874_m1); *Cart* (Mm00489086_m1); *Agrp* (Mm00475829_g1); *Npy* (Mm00445771_m1); *Pcsk1* (Mm00479023_m1); *Mcr3* (Mm00434876_s1); and *Mcr4* (Mm00457483_s1). The expression level was normalized against the housekeeping gene *Gapdh* (Mm99999915_g1). Data were analyzed using the standard curve method.

### α-MSH and AgRP immunofluorescence

Three-week-old brains derived from 10 different litters (*n* = 5 litters/maternal treatment) were dissected and fixed in 4% paraformaldehyde overnight at 4°C. Cryoprotected 3-week-old brains were frozen in smashed dry ice and sectioned using a cryostat (Leica CM 1950). Selected 20 μm-thick sections (1 out of 4 sections) throughout the PVH were used. For α-MSH staining, sections were blocked with 2% donkey serum in KPBS + 0.4% Triton X-100 for 1 h and subsequently incubated with sheep anti-α-MSH (1:750; Millipore) in blocking solution overnight at 4°C. As secondary antibody, a donkey anti-sheep Alexa Fluor 488 or 594 (1:300; Life Technologies) in KPBS + 0.4% Triton X-100 was used (2 h at room temperature). For AgRP staining, sections were blocked with 2% chicken serum in KPBS + 0.4% Triton X-100 for 1 h and subsequently incubated with rabbit anti-AgRP (1:500; Phoenix Pharmaceuticals) in blocking solution for 48 h at 4°C. As secondary antibody, a chicken anti-rabbit Alexa Fluor 488 (1:300; Life Technologies) in KPBS + 0.4% Triton X-100 was used (2 h at room temperature).

### Fiber density quantitative analysis

For quantification of neuronal fiber density staining, images of 4 representative sections throughout the PVH (bregma between −0.59 mm and −1.23 mm) from each animal were acquired using an Olympus fluorescence microscope equipped with a 20× objective. α-MSH and AgRP fiber density analyses were performed in blind conditions using FIJI (ImageJ) Launcher based on previously published reports [26,39]. Briefly, each image was binarized to compensate for differences in fluorescence intensity, specified in a random 200 × 200 μm region and skeletonized, so that each fiber segment corresponded to 1 pixel thick. The integrated intensity was then measured for each image. The total density value was obtained by the sum of all image planes analyzed.

### General behavioral procedures

Mice were acclimatized to the behavioral room for 1 h before each test. The arena was cleaned with 70% ethanol before and after each trial. Light intensity was adapted to each task. During testing, the investigator remained outside the behavioral laboratory. A video camera, positioned directly above the arena, was used to record the behavior of each animal. Videos were recorded on a computer and analyzed with a dedicated behavioral video-tracking software (SMART v3.0, Panlab). For chow studies, a total number of 6 control and 4 emulsifiers-treated litters were used. During western diet exposure, a total of 5 controls and 3 emulsifiers-treated litters were used.

### Open field test

The open field test is generally used for measuring the exploratory behavior and general activity of animals. Our protocol was based on former studies [59]. Mice were placed in the center of a dark methacrylate arena (35 × 35 × 35 cm) and allowed to freely explore it for 15 min. Light was set at low intensity (<30 lux) to avoid stress. Total distance and time spent in the corner and center of the arena were scored using SMART v3.0 software (Panlab).

### Dark-light box test

The dark-light box test assesses anxiety-like behavior. This paradigm takes advantage of the aversion of rodents to brightly illuminated areas. Our protocol was based on a previous report by Fan and colleagues [59]. The test apparatus comprised a methacrylate arena (35 × 35 × 35 cm) divided into a small dark (safe) compartment and a large strongly illuminated (200 lux; aversive) compartment. The 2 compartments were connected. Mice were positioned in the dark chamber and allowed to freely explore the 2 compartments for 5 min. Video-tracking data was analyzed to measure the time spent in each chamber and the latency to enter the illuminated area using SMART v3.0 software (Panlab).

### Novel object recognition test (NORT)

NORT is a widely validated task to evaluate recognition memory. Our protocol was based on previous reports [60]. The test was conducted in a methacrylate arena (35 × 35 × 35 cm) with low-intensity light (20 lux) environment. NORT comprises 3 phases: habituation, training, and test. During habituation (days 1 to 3), mice were allowed to freely explore the arena for 10 min. In the training period (day 4), mice were allowed to explore 2 equidistantly spaced identical objects (in the arena) for 10 min and returned to their home cages afterward. The test phase was conducted 2 h after the training phase. In this stage, one of the objects was replaced by a new one (novel object). Mice were allowed to explore the objects for 10 min. The position of the 2 items was constant across sessions. Discrimination indices were calculated as: (Time exploring novel object–Time exploring familiar object)/(Time exploring novel object + Time exploring familiar object). We also scored total exploration time (Time exploring novel object + Time exploring familiar object) and total distance traveled. Mice that exhibited freezing behavior or <5 s of exploration behavior were excluded from the analyses. Trials were video recorded and analyzed offline in a blind manner using SMART v3.0 software (Panlab). Total distance was automatically scored by the software.

### Statistical analysis

Data were expressed as mean ± SEM. Statistical analyses were performed using GraphPad Prism software (version 8.0). For dams’ experiments and offspring’s characterization at weaning (two-group one-factor comparisons) statistical analysis using a two-tailed unpaired Student’s *t* test was performed. For all adulthood studies, in order to take into account the effect of maternal programming (2 factors-1 dependent variable), statistical analysis was performed using two-way ANOVA followed by Sidak’s post hoc test. *p* < 0.05 was considered statistically significant. Symbols used were: *p < 0.05; **p < 0.01; ***p < 0.001; ****p < 0.0001. Statistical parameters can be found in the figures and their legends.

## Supporting information

S1 Table List of differentially expressed genes (DEGs) and gene ontology (GO) enriched pathway analysis of the down–regulated genes obtained from RNA sequencing of P21 male offspring from control and emulsifiers–treated dams. (XLSX)

S1 Fig Emulsifiers induce mild maternal glucose intolerance. (**A**) Experimental design of maternal emulsifier consumption highlighting the period of maternal characterization. (**B**) Daily food intake of control and emulsifier–treated dams post–weaning (*n* = 6/group). (**C**) Body weight of control and emulsifier–treated dams post–weaning (*n* = 15 CTRL and *n* = 15 Emul). (**D**) gWAT weight normalized by total body weight and represented as % of control animals of control and emulsifier dams post–weaning (*n* = 15 CTRL and *n* = 15 Emul). (**E**) GTT and (**F**) AUC of control and emulsifier–treated dams post–weaning (*n* = 15 CTRL and *n* = 15 Emul). (**G**) Fasting blood glucose levels of control and emulsifier–treated dams post–weaning (*n* = 15 CTRL and *n* = 15 Emul). (**H**) Plasma leptin levels after 6 h of fasting of control and emulsifier–treated dams post–weaning (*n* = 6 CTRL and *n* = 7 Emul). (**I**) Plasma insulin levels after 6 h of fasting of control and emulsifier–treated dams post–weaning (*n* = 7 CTRL and *n* = 7 Emul). Data in **B**, **H**, and **I** are derived from 1 single experiment. Data in **C**, **D**, **E**, **F**, and **G** are pools from 2 different experiments. Data are expressed as mean ± SEM. Statistical analysis was performed with an unpaired *t* test in **B**, **C**, **D**, **F**, **G**, **H**, and **I** and by two–way ANOVA followed by Sidak’s post hoc analysis in **E**. *p < 0.05; **p < 0.01; ***p < 0.001. The data underlying this figure can be found at DOI:10.6084/m9.figshare.22742759.
(TIF)

S2 Fig (**A**) PCA plot showing the distribution of sequenced samples (*n* = 4 CTRL and *n* = 5 Emul). (**B**) Representative 20× images of POMC neurons in the ARC of control and emulsifiers–treated male offspring at P21. (**C**) Number of POMC neurons per section of control and emulsifier offspring at P21 (*n* = 3 mice/group). (**D**) POMC neuronal area of control and emulsifier offspring at P21 (*n* = 20 neurons per animal; 3 mice/group). Data are expressed as mean ± SEM. Statistical analysis was performed by *t* test. POMC: pro–opiomelanocortin; 3V: third ventricle; ns: non–significant. The data underlying this figure can be found at DOI:10.6084/m9.figshare.22742759.
(TIF)

S3 Fig Female offspring from emulsifier–treated dams do not present metabolic impairments. (**A**) Body weight at 10 weeks of age (*n* = 5 CTRL–CTRL; *n* = 6 CTRL–Emul; *n* = 7 Emul–CTRL; *n* = 6 Emul–Emul). (**B**) Body length at 10 weeks of age (*n* = 7 CTRL–CTRL; *n* = 9 CTRL–Emul; *n* = 6 Emul–CTRL; *n* = 8 Emul–Emul). (**C**) gWAT weight normalized by total body weight and represented as % of control animals at 10 weeks of age (*n* = 5 CTRL–CTRL; *n* = 6 CTRL–Emul; *n* = 7 Emul–CTRL; *n* = 6 Emul–Emul). (**D**) GTT and (**E**) AUC (*n* = 13 CTRL–CTRL; *n* = 14 CTRL–Emul; *n* = 15 Emul–CTRL; *n* = 15 Emul–Emul) at 10 weeks of age. (**F**) ITT and (**G**) AUC (*n* = 7 CTRL–CTRL; *n* = 10 CTRL–Emul; *n* = 14 Emul–CTRL; *n* = 12 Emul–Emul) at 10 weeks of age. (**H**) Six–hour fasting blood glucose levels at 10 weeks of age (*n* = 5 CTRL–CTRL; *n* = 6 CTRL–Emul; *n* = 7 Emul–CTRL; *n* = 6 Emul–Emul). (**I**) Plasma insulin levels after 6 h of fasting at 10 weeks of age (*n* = 4 CTRL–CTRL; *n* = 5 CTRL–Emul; *n* = 5 Emul–CTRL; *n* = 6 Emul–Emul). (**J**) Plasma leptin levels after 6 h of fasting at 10 weeks of age (*n* = 5 CTRL–CTRL; *n* = 5 CTRL–Emul; *n* = 7 Emul–CTRL; *n* = 6 Emul–Emul). Data in **A**, **B**, **C**, **H**, **I**, and **J** are derived from 1 single experiment. Data in **D**, **E**, **F**, and **G** are pools from 2 different experiments. Data are expressed as mean ± SEM. Statistical analysis was performed by two–way ANOVA followed by Sidak’s post hoc analysis. The data underlying this figure can be found at DOI:10.6084/m9.figshare.22742759.
(TIF)

S4 Fig Male offspring from emulsifier–treated dams respond normally to leptin. (**A**) Food intake of males at 20 weeks of age (*n* = 9 CTRL–CTRL; *n* = 9 CTRL–Emul; *n* = 6 Emul–CTRL; *n* = 5 Emul–Emul). (**B**) Food intake of females at 20 weeks of age (*n* = 9 CTRL–CTRL; *n* = 12 CTRL–Emul; *n* = 6 Emul–CTRL; *n* = 8 Emul–Emul). (**C**) Average overnight food intake of males after vehicle (Vh) (*n* = 8 CTRL–CTRL; *n* = 8 CTRL–Emul; *n* = 5 Emul–CTRL; *n* = 5 Emul–Emul) or leptin (Lep) (*n* = 8 CTRL–CTRL; *n* = 8 CTRL–Emul; *n* = 5 Emul–CTRL; *n* = 5 Emul–Emul) injection at 20 weeks of age. (**D**) Overnight body weight after vehicle (*n* = 8 CTRL–CTRL; *n* = 8 CTRL–Emul; *n* = 5 Emul–CTRL; *n* = 5 Emul–Emul) or leptin (*n* = 8 CTRL–CTRL; *n* = 8 CTRL–Emul; *n* = 5 Emul–CTRL; *n* = 5 Emul–Emul) injection in males at 20 weeks of age. Data in **A**, **B**, **C**, and **D** are pools from 2 different experiments. Data are expressed as mean ± SEM. Statistical analysis was performed by two–way ANOVA followed by Sidak’s post hoc analysis in **A**, **B**, and by *t* test in **C** and **D**. ns: not significant; *p < 0.05; **p < 0.01. The data underlying this figure can be found at DOI:10.6084/m9.figshare.22742759.
(TIF)

S5 Fig Maternal emulsifier consumption induces anxiety–related states in male offspring. (**A–D**) Open field performance in 9–week–old male (**A** and **B**) (*n* = 9 CTRL–CTRL; *n* = 9 CTRL–Emul; *n* = 6 Emul–CTRL; *n* = 6 Emul–Emul) and female (**C** and **D**) (*n* = 9 CTRL–CTRL; *n* = 8 CTRL–Emul; *n* = 5 Emul–CTRL; *n* = 5 Emul–Emul) offspring born of control and emulsifier–exposed mothers, including time spent per zone (**A** and **C**) and total distance traveled (**B** and **D**). (**E, F**) Time spent in the light compartment during the dark–light box test in 9–week–old male (**E**) (*n* = 9 CTRL–CTRL; *n* = 9 CTRL–Emul; *n* = 6 Emul–CTRL; *n* = 6 Emul–Emul) and female (**F**) (*n* = 9 CTRL–CTRL; *n* = 8 CTRL–Emul; *n* = 5 Emul–CTRL; *n* = 5 Emul–Emul) offspring born of control and emulsifier–exposed mothers. (**G–J**) Short–term memory parameters in 10–week–old male (**G** and **H**) (*n* = 9 CTRL–CTRL; *n* = 9 CTRL–Emul; *n* = 4 Emul–CTRL; *n* = 6 Emul–Emul) and female (**I** and **J**) (*n* = 7 CTRL–CTRL; *n* = 7 CTRL–Emul; *n* = 3 Emul–CTRL; *n* = 4 Emul–Emul) offspring born of control and emulsifier–exposed mothers, including discrimination index (**G** and **I**) and exploratory time (**H** and **J**). Data are derived from 1 single experiment. Data are expressed as mean ± SEM. Statistical analysis was performed by two–way ANOVA followed by Sidak’s post hoc analysis. *p < 0.05. The data underlying this figure can be found at DOI:10.6084/m9.figshare.22742759.
(TIF)

## Abbreviations

AgRP: agouti-related peptide
AUC: area under the curve
CMC: carboxymethyl cellulose
DEG: differentially expressed gene
FAO: Food and Agriculture Organization
GTT: glucose tolerance test
gWAT: gonadal white adipose tissue
IP: intraperitoneally
MBH: mediobasal hypothalamus
NORT: novel object recognition test
P80: polysorbate 80
PCA: principal component analysis
POMC: pro-opiomelanocortin
PVH: paraventricular nucleus of the hypothalamus
qPCR: quantitative polymerase chain reaction
RIN: RNA integrity number
T2D: type 2 diabetes
UPF: ultra-processed food
WD: western-style diet
WHO: World Health Organization
