# Supplementary figures and images for: Maternal emulsifier consumption programs offspring metabolic and neuropsychological health in mice
Source: PLoS Biol. 2023 Aug 24;21(8):e3002171. doi: 10.1371/journal.pbio.3002171 (PMC10449393; doi:10.1371/journal.pbio.3002171)

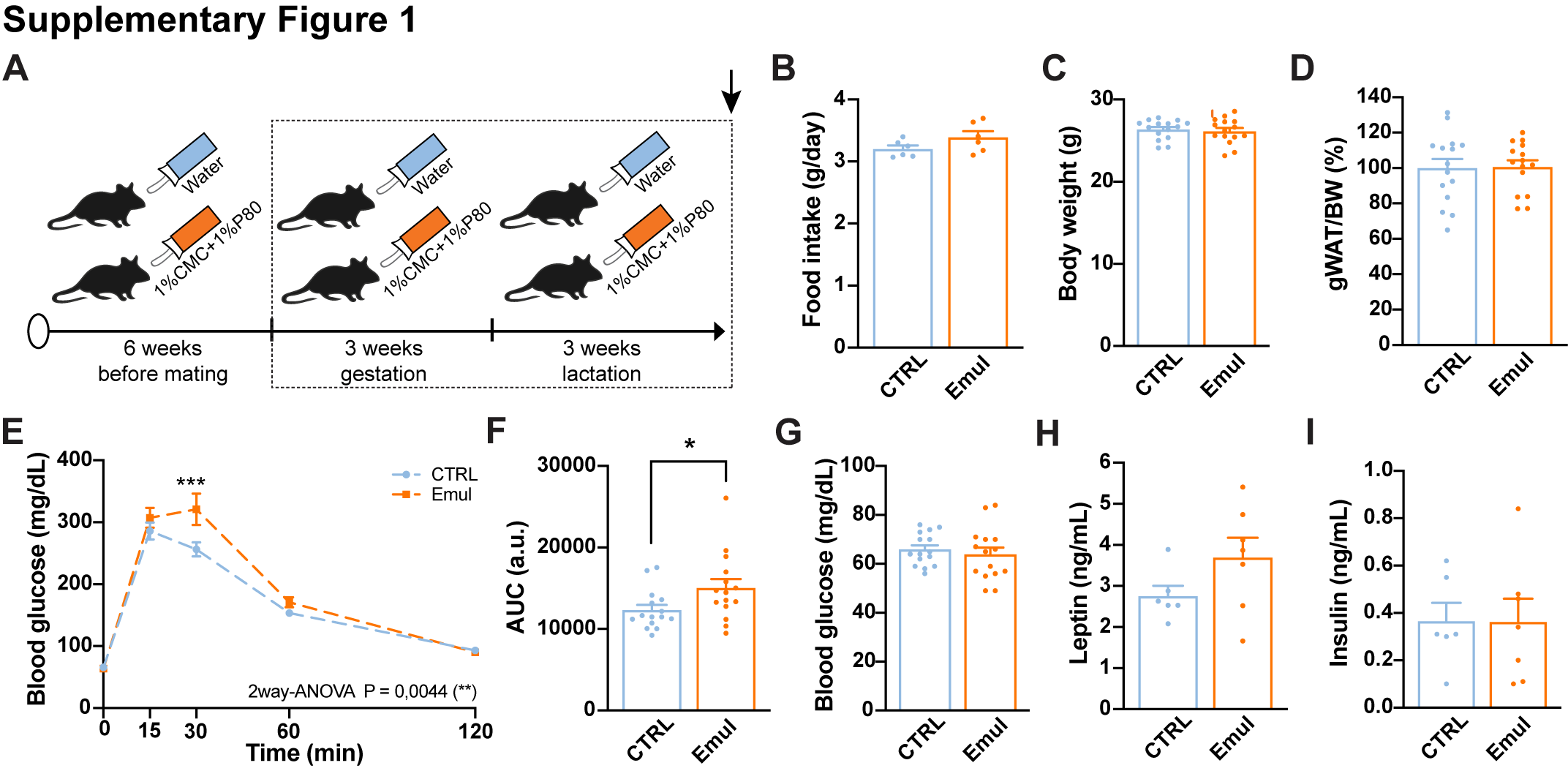

Supplement: S1 Fig — (A) Experimental design of maternal emulsifier consumption highlighting the period of maternal characterization. (B) Daily food intake of control and emulsifier–treated dams post–weaning (n = 6/group). (C) Body weight of control and emulsifier–treated dams post–weaning (n = 15 CTRL and n = 15 Emul). (D) gWAT weight normalized by total body weight and represented as % of control animals of control and emulsifier dams post–weaning (n = 15 CTRL and n = 15 Emul). (E) GTT and (F) AUC of control and emulsifier–treated dams post–weaning (n = 15 CTRL and n = 15 Emul). (G) Fasting blood glucose levels of control and emulsifier–treated dams post–weaning (n = 15 CTRL and n = 15 Emul). (H) Plasma leptin levels after 6 h of fasting of control and emulsifier–treated dams post–weaning (n = 6 CTRL and n = 7 Emul). (I) Plasma insulin levels after 6 h of fasting of control and emulsifier–treated dams post–weaning (n = 7 CTRL and n = 7 Emul). Data in B, H, and I are derived from 1 single experiment. Data in C, D, E, F, and G are pools from 2 different experiments. Data are expressed as mean ± SEM. Statistical analysis was performed with an unpaired t test in B, C, D, F, G, H, and I and by two–way ANOVA followed by Sidak’s post hoc analysis in E. *p < 0.05; **p < 0.01; ***p < 0.001. The data underlying this figure can be found at DOI:10.6084/m9.figshare.22742759. (TIF) [file pbio.3002171.s002.tif]

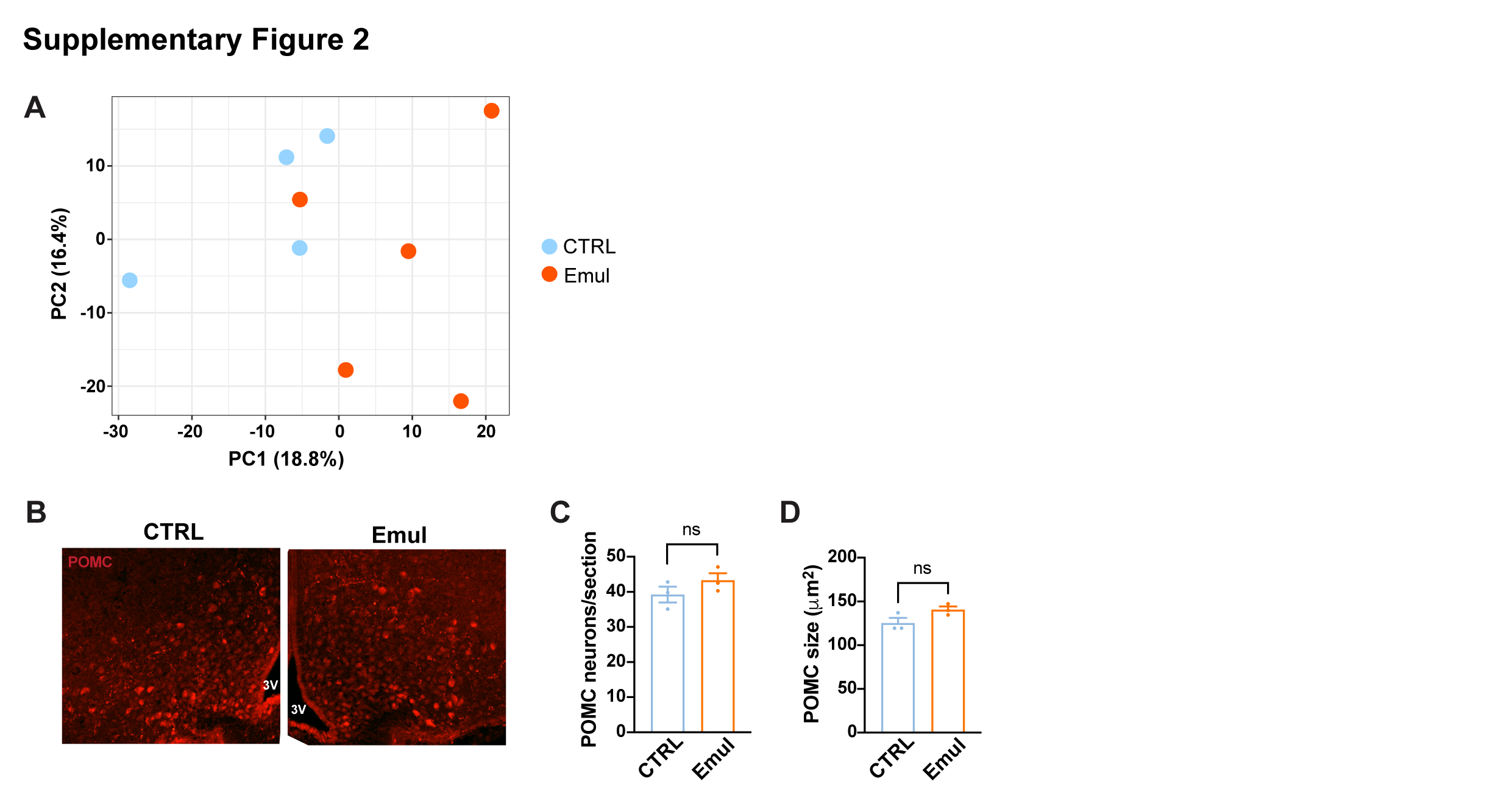

Supplement: S2 Fig — (A) PCA plot showing the distribution of sequenced samples (n = 4 CTRL and n = 5 Emul). (B) Representative 20× images of POMC neurons in the ARC of control and emulsifiers–treated male offspring at P21. (C) Number of POMC neurons per section of control and emulsifier offspring at P21 (n = 3 mice/group). (D) POMC neuronal area of control and emulsifier offspring at P21 (n = 20 neurons per animal; 3 mice/group). Data are expressed as mean ± SEM. Statistical analysis was performed by t test. POMC: pro–opiomelanocortin; 3V: third ventricle; ns: non–significant. The data underlying this figure can be found at DOI:10.6084/m9.figshare.22742759. (TIF) [file pbio.3002171.s003.tif]

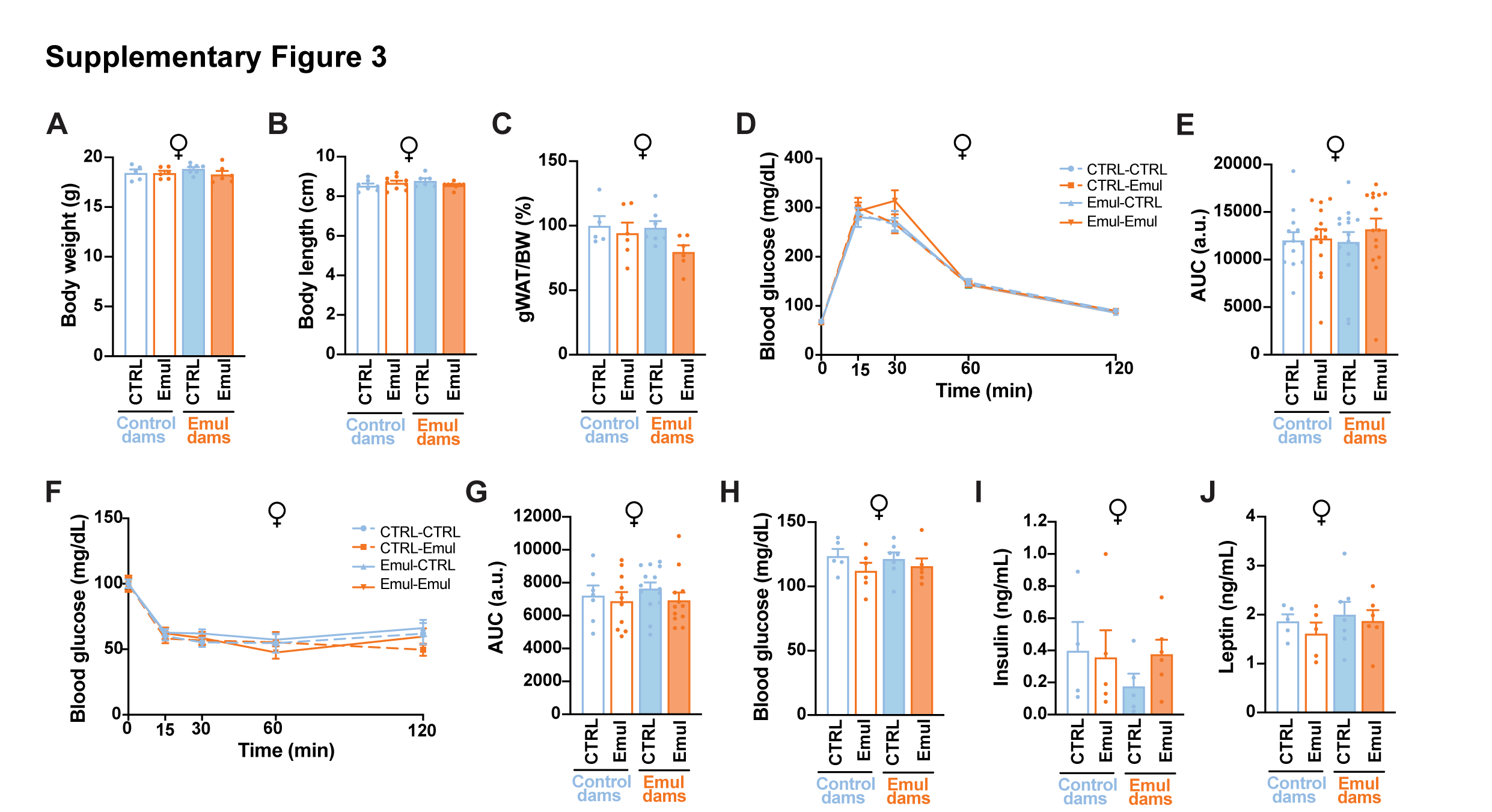

Supplement: S3 Fig — (A) Body weight at 10 weeks of age (n = 5 CTRL–CTRL; n = 6 CTRL–Emul; n = 7 Emul–CTRL; n = 6 Emul–Emul). (B) Body length at 10 weeks of age (n = 7 CTRL–CTRL; n = 9 CTRL–Emul; n = 6 Emul–CTRL; n = 8 Emul–Emul). (C) gWAT weight normalized by total body weight and represented as % of control animals at 10 weeks of age (n = 5 CTRL–CTRL; n = 6 CTRL–Emul; n = 7 Emul–CTRL; n = 6 Emul–Emul). (D) GTT and (E) AUC (n = 13 CTRL–CTRL; n = 14 CTRL–Emul; n = 15 Emul–CTRL; n = 15 Emul–Emul) at 10 weeks of age. (F) ITT and (G) AUC (n = 7 CTRL–CTRL; n = 10 CTRL–Emul; n = 14 Emul–CTRL; n = 12 Emul–Emul) at 10 weeks of age. (H) Six–hour fasting blood glucose levels at 10 weeks of age (n = 5 CTRL–CTRL; n = 6 CTRL–Emul; n = 7 Emul–CTRL; n = 6 Emul–Emul). (I) Plasma insulin levels after 6 h of fasting at 10 weeks of age (n = 4 CTRL–CTRL; n = 5 CTRL–Emul; n = 5 Emul–CTRL; n = 6 Emul–Emul). (J) Plasma leptin levels after 6 h of fasting at 10 weeks of age (n = 5 CTRL–CTRL; n = 5 CTRL–Emul; n = 7 Emul–CTRL; n = 6 Emul–Emul). Data in A, B, C, H, I, and J are derived from 1 single experiment. Data in D, E, F, and G are pools from 2 different experiments. Data are expressed as mean ± SEM. Statistical analysis was performed by two–way ANOVA followed by Sidak’s post hoc analysis. The data underlying this figure can be found at DOI:10.6084/m9.figshare.22742759. (TIF) [file pbio.3002171.s004.tif]

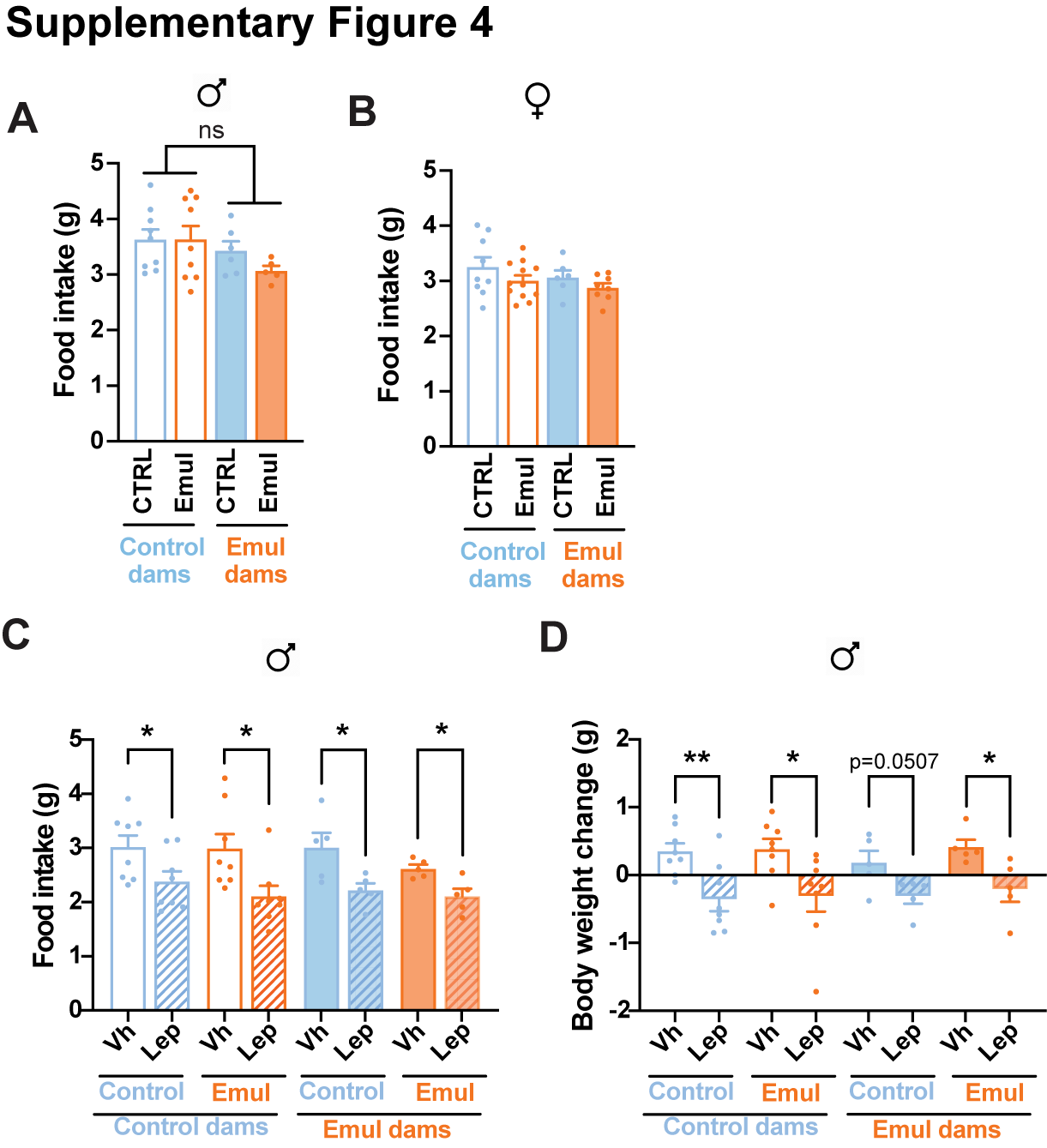

Supplement: S4 Fig — (A) Food intake of males at 20 weeks of age (n = 9 CTRL–CTRL; n = 9 CTRL–Emul; n = 6 Emul–CTRL; n = 5 Emul–Emul). (B) Food intake of females at 20 weeks of age (n = 9 CTRL–CTRL; n = 12 CTRL–Emul; n = 6 Emul–CTRL; n = 8 Emul–Emul). (C) Average overnight food intake of males after vehicle (Vh) (n = 8 CTRL–CTRL; n = 8 CTRL–Emul; n = 5 Emul–CTRL; n = 5 Emul–Emul) or leptin (Lep) (n = 8 CTRL–CTRL; n = 8 CTRL–Emul; n = 5 Emul–CTRL; n = 5 Emul–Emul) injection at 20 weeks of age. (D) Overnight body weight after vehicle (n = 8 CTRL–CTRL; n = 8 CTRL–Emul; n = 5 Emul–CTRL; n = 5 Emul–Emul) or leptin (n = 8 CTRL–CTRL; n = 8 CTRL–Emul; n = 5 Emul–CTRL; n = 5 Emul–Emul) injection in males at 20 weeks of age. Data in A, B, C, and D are pools from 2 different experiments. Data are expressed as mean ± SEM. Statistical analysis was performed by two–way ANOVA followed by Sidak’s post hoc analysis in A, B, and by t test in C and D. ns: not significant; *p < 0.05; **p < 0.01. The data underlying this figure can be found at DOI:10.6084/m9.figshare.22742759. (TIF) [file pbio.3002171.s005.tif]

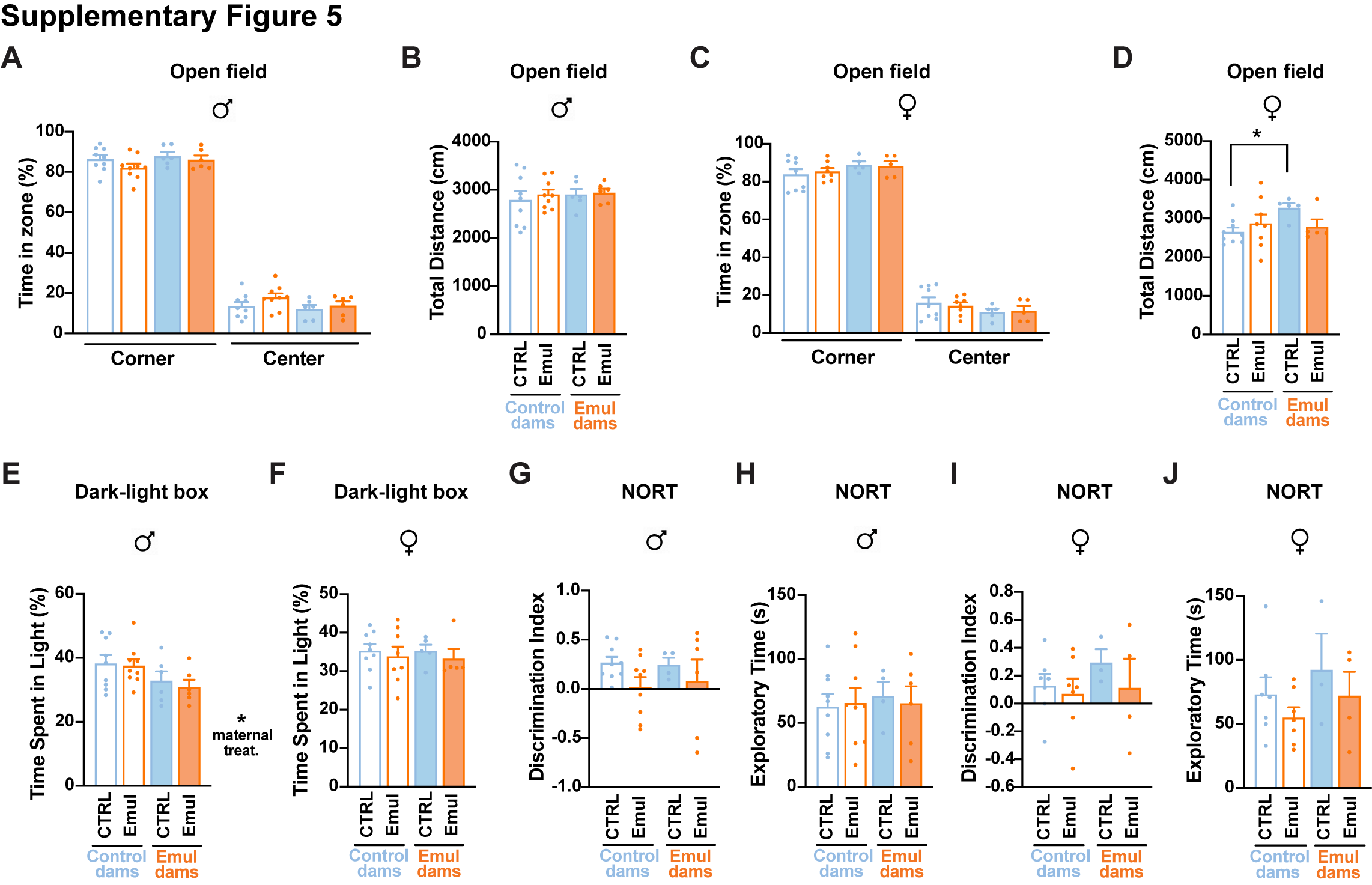

Supplement: S5 Fig — (A–D) Open field performance in 9–week–old male (A and B) (n = 9 CTRL–CTRL; n = 9 CTRL–Emul; n = 6 Emul–CTRL; n = 6 Emul–Emul) and female (C and D) (n = 9 CTRL–CTRL; n = 8 CTRL–Emul; n = 5 Emul–CTRL; n = 5 Emul–Emul) offspring born of control and emulsifier–exposed mothers, including time spent per zone (A and C) and total distance traveled (B and D). (E, F) Time spent in the light compartment during the dark–light box test in 9–week–old male (E) (n = 9 CTRL–CTRL; n = 9 CTRL–Emul; n = 6 Emul–CTRL; n = 6 Emul–Emul) and female (F) (n = 9 CTRL–CTRL; n = 8 CTRL–Emul; n = 5 Emul–CTRL; n = 5 Emul–Emul) offspring born of control and emulsifier–exposed mothers. (G–J) Short–term memory parameters in 10–week–old male (G and H) (n = 9 CTRL–CTRL; n = 9 CTRL–Emul; n = 4 Emul–CTRL; n = 6 Emul–Emul) and female (I and J) (n = 7 CTRL–CTRL; n = 7 CTRL–Emul; n = 3 Emul–CTRL; n = 4 Emul–Emul) offspring born of control and emulsifier–exposed mothers, including discrimination index (G and I) and exploratory time (H and J). Data are derived from 1 single experiment. Data are expressed as mean ± SEM. Statistical analysis was performed by two–way ANOVA followed by Sidak’s post hoc analysis. *p < 0.05. The data underlying this figure can be found at DOI:10.6084/m9.figshare.22742759. (TIF) [file pbio.3002171.s006.tif]
